# Revisiting chloroplast genomic landscape and annotation towards comparative chloroplast genomes of Rhamnaceae

**DOI:** 10.1186/s12870-023-04074-5

**Published:** 2023-01-28

**Authors:** Kwanjeera Wanichthanarak, Intawat Nookaew, Phongthana Pasookhush, Thidathip Wongsurawat, Piroon Jenjaroenpun, Namkhang Leeratsuwan, Songsak Wattanachaisaereekul, Wonnop Visessanguan, Yongyut Sirivatanauksorn, Narong Nuntasaen, Chutima Kuhakarn, Vichai Reutrakul, Pravech Ajawatanawong, Sakda Khoomrung

**Affiliations:** 1 Metabolomics and Systems Biology, Department of Biochemistry, Faculty of Medicine Siriraj Hospital, Mahidol University, Bangkok, 10700 Thailand; 2 Siriraj Metabolomics and Phenomics Center, Faculty of Medicine Siriraj Hospital, Mahidol University, Bangkok, 10700 Thailand; 3 Department of Biomedical Informatics, College of Medicine, University of Arkansas for Medical Sciences, Little Rock, AR 72205 USA; 4 Division of Bioinformatics and Data Management for Research, Faculty of Medicine Siriraj Hospital, Mahidol University, Bangkok, 10700 Thailand; 5 Department of Biology, Faculty of Science, Mahidol University, Bangkok, 10400 Thailand; 6 School of Food industry, King Mongkut’s Institute of Technology Ladkrabang, Bangkok, 10520 Thailand; 7 Functional Ingredients and Food Biotechnology Research Unit, National Center for Genetic Engineering and Biotechnology (BIOTEC), Phathumthani, 12120 Thailand; 8 Department of Chemistry and Center of Excellence for Innovation in Chemistry (PERCH-CIC), Faculty of Science, Mahidol University, Bangkok, 10400 Thailand; 9 Department of National Parks, Wildlife and Plant Conservation, Ministry of Natural Resources and Environment, Bangkok, 10900 Thailand

**Keywords:** *Ventilago harmandiana*, Rhamnaceae, Chloroplast genome, Plant genomics, Natural product, Genome assembly, Genome annotation, Oxford Nanopore technologies

## Abstract

**Background:**

Massive parallel sequencing technologies have enabled the elucidation of plant phylogenetic relationships from chloroplast genomes at a high pace. These include members of the family Rhamnaceae. The current Rhamnaceae phylogenetic tree is from 13 out of 24 Rhamnaceae chloroplast genomes, and only one chloroplast genome of the genus Ventilago is available. Hence, the phylogenetic relationships in Rhamnaceae remain incomplete, and more representative species are needed.

**Results:**

The complete chloroplast genome of *Ventilago harmandiana* Pierre was outlined using a hybrid assembly of long- and short-read technologies. The accuracy and validity of the final genome were confirmed with PCR amplifications and investigation of coverage depth. Sanger sequencing was used to correct for differences in lengths and nucleotide bases between inverted repeats because of the homopolymers. The phylogenetic trees reconstructed using prevalent methods for phylogenetic inference were topologically similar. The clustering based on codon usage was congruent with the molecular phylogenetic tree. The groups of genera in each tribe were in accordance with tribal classification based on molecular markers. We resolved the phylogenetic relationships among six *Hovenia species*, three *Rhamnus species*, and two *Ventilago species*. Our reconstructed tree provides the most complete and reliable low-level taxonomy to date for the family Rhamnaceae. Similar to other higher plants, the RNA editing mostly resulted in converting serine to leucine. Besides, most genes were subjected to purifying selection. Annotation anomalies, including indel calling errors, unaligned open reading frames of the same gene, inconsistent prediction of intergenic regions, and misannotated genes, were identified in the published chloroplast genomes used in this study. These could be a result of the usual imperfections in computational tools, and/or existing errors in reference genomes. Importantly, these are points of concern with regards to utilizing published chloroplast genomes for comparative genomic analysis.

**Conclusions:**

In summary, we successfully demonstrated the use of comprehensive genomic data, including DNA and amino acid sequences, to build a reliable and high-resolution phylogenetic tree for the family Rhamnaceae. Additionally, our study indicates that the revision of genome annotation before comparative genomic analyses is necessary to prevent the propagation of errors and complications in downstream analysis and interpretation.

**Supplementary Information:**

The online version contains supplementary material available at 10.1186/s12870-023-04074-5.

## Background

Advances in high-throughput sequencing technologies have enabled sequencing of whole chloroplast genomes of numerous plant species on a massive scale. This has led to the broad use of chloroplast genomes in plant evolutionary studies to achieve phylogenetic resolution at the genus and species levels. The key benefits of using chloroplast genomes for phylogenetic research include their haploid state, uniparental inheritance, and high conservation of quadripartite structures. Here, large single-copy (LSC) and small single-copy (SSC) regions are separated by two inverted repeats (IRs: IRa and IRb), whose gene content and order are well conserved [1, 2]. Although variations in chloroplast genomes (for example, genome inversions, deletions, and insertions) rarely occur, small changes in their nature are remarkably informative for phylogenetic reconstruction. Moreover, the average size of chloroplast sequences (120,000–160,000 bp) and the average number of genes (110–130 genes) provide an adequate degree of amplitude and complexity to contain structural and point mutations for extensive evolutionary classification [2]. Whole chloroplast genomes have therefore been successfully used to resolve the phylogenies of numerous plant species [3–6].

Short-read sequencing technologies, such as Illumina platforms, have been widely used in chloroplast genome studies because of their ability to rapidly produce large, high-accuracy datasets at low cost [7]. However, genome assembly, particularly across two IRs, is challenging when only short reads are used, because the approximate sizes of IRs (10–30 kb [7]) are typically larger than the generated read length (50–400 bp [7]). Recent single-molecule sequencing platforms, such as Oxford Nanopore Technologies (ONT) and Pacific Biosciences (PacBio), can reportedly produce a single read of longer than 10 kb to 200 kb for a large chloroplast section [7, 8]. However, when compared to short-read technologies, the per-base error rate of long reads is relatively high (5–10% [7]). Thus, a combination of long- and short-read datasets is a promising solution that can be apply for chloroplast genomes. It has been reported that approximately 20x coverage of long- and short-read hybrid assemblies can generate a single contig of the entire chloroplast genome [7]. Here, long reads are useful for elucidating chloroplast structure, whereas the few fragmented contigs and sequencing errors can later be polished using short reads owing to their higher accuracy [7]. This is a good practice to obtain high quality genome sequences [9]. Once chloroplast genomes are assembled, they are annotated to identify fundamental features such as protein-coding sequences, tRNA genes, rRNA genes, intergenic regions, and IRs. This process is usually performed using automated annotation tools, such as DOGMA [10], CpGAVAS [11] and GeSeq [12]. However, most tools rely on existing genomes deposited in databases (such as GenBank [13]), which are not completely correct [14]. Therefore, subsequent curation by domain experts is a prerequisite before downstream analysis. Using Rhamnaceae as an example, our study revealed the significance of revisiting genome annotation prior to comparative genomic analysis.

Rhamnaceae is a plant family comprising approximately 55 genera and 950 species, including trees, shrubs, climbers, and herbs [15–17]. It belongs to the order Rosales, and several members of the family Rhamnaceae are of great economic and medicinal values. Chinese jujube (*Ziziphus jujuba* Mill) and Indian jujube (*Ziziphus mauritiana)* fruits are economically important [18]. Members of genus Hovenia are used for making fine furniture and musical instruments [18–20]. Other species, such as *Hovenia dulcis* [21], Ventilago species [22, 23] and Ziziphus species [24], possess medicinal properties. Naturally, Rhamnaceae species reside in many different habitats, ranging from tropical rain forests to moderately arid regions and from sea level to treelines. This family appears to have a high morphological and genetic diversity [25]. The traditional classification of Rhamnaceae tribes relies on morphological features such as floral traits, fruit characteristics, and a few marker genes. Until recently, the basis of taxonomic classification has shifted towards phylogenomic analysis of complete chloroplast genomes. Of over 5300 plant chloroplast genomes, only 24 complete genomes from seven genera of the family Rhamnaceae, namely Berchemia, Berchemiella, Hovenia, Rhamnus, Spyridium, Ventilago, and Ziziphus, have been published (National Center for Biotechnology Information (NCBI) database on July 2021). Notably, only one genome of the Ventilago species from China, *Ventilago leiocarpa* Benth (*V. leiocarpa*), is available. In particular, the current Rhamnaceae phylogenetic tree constructed by Wang et al. (2021) contains 13 unique species from six genera (Berchemia, Berchemiella, Hovenia, Rhamnus, Spyridium, and Ziziphus), except for Ventilago [26]. Another tree constructed by Lu et al. (2021) includes *V. leiocarpa* [27]. However, only seven Rhamnaceae species have been analyzed. Hence, the phylogenetic relationships between Ventilago and other Rhamnaceae organisms remain inconclusive and require more representative species. *Ventilago harmandiana* Pierre (*V. harmandiana*) is a plant species of the genus Ventilago within the family Rhamnaceae, which is found across Asia, including China, India, Malaysia, Singapore, and Thailand [22, 28, 29]. Indeed, Ventilago consists of over 40 species, including medicinal plants with numerous therapeutic properties, such as cytotoxicity to cancer cells [22], antimicrobial activity [30] and anti-inflammatory effects, as examined in animal models [31, 32]. Our previous study indicated that pyranonaphthoquinones from the heartwood extract of *V. harmandiana* are promising compounds with cytotoxic and anti-inflammatory properties [23]. Although it shows promise with regards to complementary or alternative therapy, it is yet to be investigated on a genomic level.

To extend the taxonomic coverage of the family Rhamnaceae, we reported the complete chloroplast genome of *V. harmandiana* using a hybrid assembly of ONT long reads and Illumina HiSeq short reads. We clustered Rhamnaceae members based on their codon usage and reconstructed the phylogenetic tree at low taxonomic levels using all published Rhamnaceae chloroplast genomes, along with the newly sequenced chloroplast genome of *V. harmandiana*. Notably, short-read sequencing technologies, such as Illumina HiSeq and MiSeq, are the primary platforms for generating DNA sequences for published Rhamnaceae chloroplast genomes. Moreover, there are differences in assembly techniques, annotation tools, choices of reference genomes, and hidden errors in computational prediction. Hence, all chloroplast genomes in this study underwent extensive curation, which included revision of gene locations, correction of gene sequences, and re-annotation of missing genes prior to comparative sequence analyses. This was done to ensure that the newly sequenced chloroplast genome of *V. harmandiana* and the reconstructed phylogenetic tree of Rhamnaceae were of high quality.

## Results

### *V. Harmandiana* chloroplast genome structure and validation

**Fig. 1:**
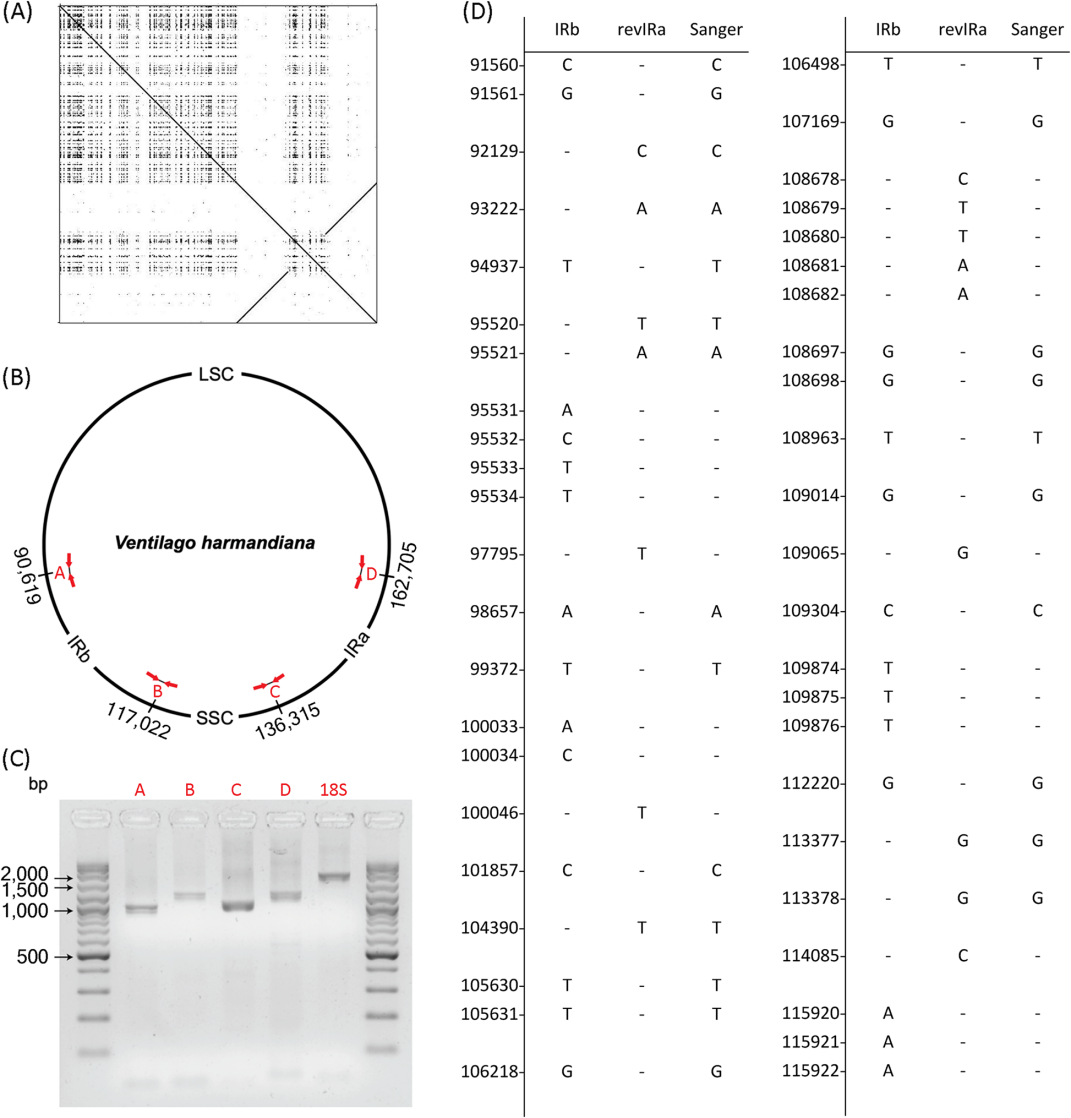
*V. harmandiana* chloroplast genome assembly and correction. **A** Identification of IRs in the chloroplast genome by dot plot. **B** A quadripartite structure of *V. harmandiana* chloroplast. There are four IR boundaries of A, B, C and D, each marked with the chloroplast genome position. An amplicon covering each boundary is indicated by the red arrows. Boundary A divides LSC and IRb. Boundary B and Boundary C separate SSC from IRb and IRa, respectively. Boundary D partitions IRa and LSC. Table S1 provides primer details, in which the primer set VharA to VharD are for the validation of the IR boundaries. **C** Validation of the IR boundaries by PCR amplification of regions spanning over the four IR boundaries. Approximate sizes of amplicons in each boundary region are 951, 1189, 1035 and 1193 nt, respectively. **D** Correction of mismatched nucleotide bases between IRs. All 45 mismatched bases and the correct DNA bases determined by Sanger sequencing are illustrated. IRb base position is uses as reference. The primer set VharIR1 to VharIR14 are to determine mismatched nucleotide bases between IRs (Additional file 2 Table S1). Large single-copy (LSC); inverted repeats (IRs), reversed inverted repeat (revIR) and small single-copy (SSC)

A total of 11,004 raw long reads with an average length of 4231 bp and 636.3 million raw short reads with an average length of approximately 150 bp were generated from the ONT and Illumina HiSeq platforms, respectively. Initially, the size of the draft chloroplast genome based on the hybrid assembly using the long- and short-read datasets was 162,893 bp. A dot plot of the *V. harmandiana* chloroplast genome illustrates the presence of a quadripartite structure with the two IRs (Fig. 1A). However, no single ONT read fully covered either IR region. In addition, the sequence length of IRs was 13 bp different, and there were 45 mismatched nucleotide bases.

To ensure the correct assembly of the quadripartite structure of the *V. harmandiana* chloroplast genome, PCR amplification of DNA regions spanning the four IR boundaries was performed (see Additional file 2 Table S1 for primer sequences). Boundary A separates LSC and IRb, Boundary B separates IRb and SSC, Boundary C separates SSC and IRa, and Boundary D separates IRa and LSC (Fig. 1B). The approximate sizes of the amplicons in each DNA region were 951, 1189, 1035, and 1193 nucleotides (nt), respectively (Fig. 1C). Furthermore, the number of spanning reads in the four DNA regions was determined (Additional file 1 Fig. S1). There were only two long reads of size 21,340 and 33,928 nt, spanning across the SSC. The overall read length, which covered the four IR boundaries, varied. However, the number of spanning reads in each region did not differ significantly. In total, there were 252, 256, 244, and 226 reads spanning boundaries A, B, C, and D, respectively. In particular, most reads were spread within 3000 nt from the borders, consisting of 216, 228, 202, and 192 reads in boundaries A, B, C, and D, respectively. Reads longer than 3000 nt accounted for approximately 10% of the total reads (Boundary A = 36, B = 28, C = 42, and D = 34 reads). The longest read for each boundary was 8934, 13,250, 10,159, and 12,531 nt in length for boundaries A, B, C and D, respectively.

The 45 mismatched nucleotide bases between the IRs were then corrected using sequences obtained from Sanger sequencing (Fig. 1D). At this step, the chloroplast genome size was 162,898 bp. The lengths of both IRs were identical, with a size of 26,399 bp. Table 1 lists the 134 genes, including 89 protein-coding genes, 37 tRNA genes, and 8 rRNA genes, identified by automated gene prediction.

**Table 1:** *V. harmandiana* chloroplast genome characteristic

Characteristic	Detail
Chromosome	Single-circular
Size (bp)	162,904
GC content (%)	36.6
Number of genes	134
LSC region	82
SSC region	12
IR regions	40
Protein-coding genes	89
tRNA genes	37
rRNA genes	8

### Revision of published chloroplast genomes: a case study in Rhamnaceae

Among the 24 Rhamnaceae chloroplast genomes from the NCBI database, we noticed that out of the total number of genes (between 121 and 131 genes), protein-coding genes (between 78 and 86 genes), and tRNA genes (between 34 and 37 genes) varied notably (Additional file 2 Table S2). In particular, the numbers of genes within the same species, such as *B. wilsonii*, *H. acerba*, *H. dulcis*, and *Z. jujuba*, were different. Moreover, seven protein-coding genes were found to differ between *V. harmandiana* and *V. leiocarpa* MT974496. We suspect that this was possibly a result of the usual imperfection in computational predictions [33], as well as mis-assembly. Therefore, all the chloroplast genomes in this study were extensively refined before further comparative genomic analysis. This was done to avoid propagation of errors that were inherited from the automated genome assemblies and annotation pipelines.

First, we identified annotation anomalies in the 26 published chloroplast genomes (Additional file 2 Table S3). These included unresolved open reading frames, unaligned coding sequences, and missing genes. *Arabidopsis thaliana* NC_000932 was used as a reference genome to manually correct gene locations, including for example, *accD*, some of the NADH dehydrogenase and photosystem genes, and unaligned gene sequences like *psbL* and *rpl36*. A missing gene was identified by local alignment with the coding sequence of the closest organism, that is, the same species or genus. Overall, the most missing genes were those in the IRa region, and the least missing ones were those in the SSC region. Most gene corrections were made to the *B. wilsonii* NC_043912 chloroplast genome, whereas the *H. acerba* MN794429, *H. dulcis* MT225403, and *H. trichocarpa* chloroplast genomes contained the highest number of missing genes.

**Fig. 2:**
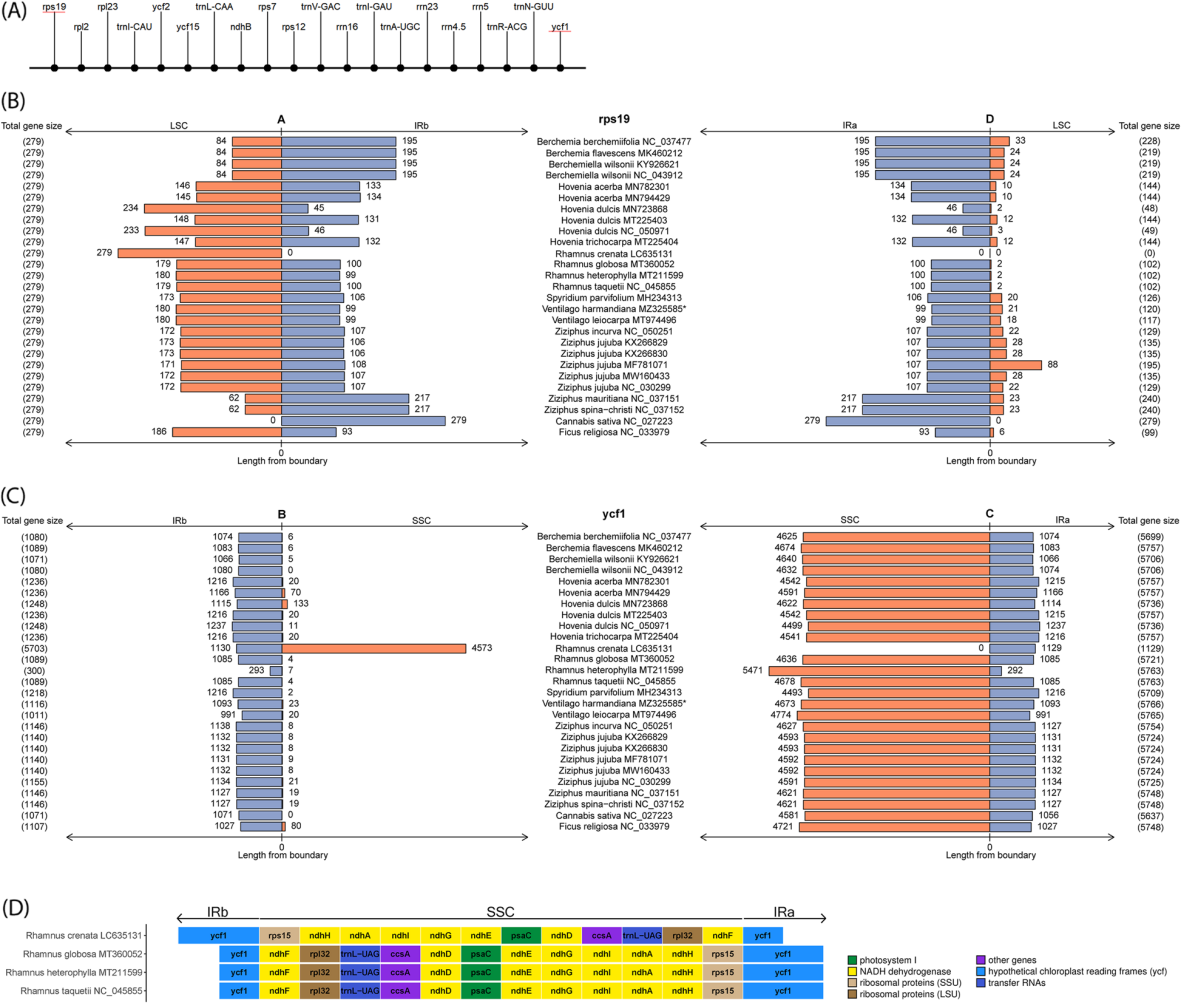
Comparative gene structure and arrangement in IR regions of Rhamnaceae, *C. sativa* and *F. religios*. **A** Consensus gene arrangement within an IRb and a reversed IRa. The *rps19* and *ycf1* genes (red underline) are located at the borders of IR regions, see Additional file 1 Fig. S2 for gene arrangement in each organism. **B** Segments of *rps19* in LSC (orange) and IR regions (blue), and **C** Segments of *ycf1* in SSC (orange) and IR regions (blue). Each bar chart shows the sizes of a gene (bp) in each region calculated from the IR boundary. Total gene sizes are shown in the parenthesis. IR boundaries (A, B, C and D) are defined as in Fig. 1. The outgroup of phylogenetic analysis includes *C. sativa* NC_027223 and *F. religiosa* NC_033979. **D** Assembly error in SSC of *R. crenata* LC635131. Genes are color coded by functional categories

The genes of the IRs were of particular interest. A comparison of gene content and arrangement in the IRs among all organisms revealed that they were highly conserved (Fig. 2A and Additional file 1 Fig. S2). In total, there were 20 copies of genes in the IRs. However, a complete collection of these genes has only been reported in the chloroplast genomes of *B. wilsonii* NC_043912 and *V. harmandiana*. In this study, the loss of a *rps12* copy from the *Z. jujuba* MF781071 and *F. religiosa* chloroplast genomes was recovered by its counterpart. The absence of both copies of *trnN-GUU* in the *R. crenata* chloroplast genome was addressed by re-annotation using *trnN-GUU* of *R. globosa* chloroplast genome as a reference (Additional file 1 Fig. S2). *rps19*, *ycf1*, and *ycf15* were absent in most organisms. Especially, *rps19* was missing from IRa. The *ycf1* gene was not annotated in IRb, whereas *ycf15* copies in IRs were not reported in many organisms in this study.

### Re-annotation and analysis of *rps19* and *ycf1* genes

In general, *rps19* and *ycf1* are located at the borders of the IR regions. In organisms such as *C. sativa*, all *rps19* genes were found in IRb and IRa. The entire *rps19* gene was found in the LSC of the *R. crenata* chloroplast genome, except for its copy in IRa. In other organisms, the IR boundaries were positioned in the coding regions of *rps19* and *ycf1*. In other words, segments of *rps19* spanned the LSC (Fig. 2B) and those of *ycf1* were integrated into the SSC (Fig. 2C). In accordance with other studies, the contracting or expanding incidence of IR boundaries from or into adjacent single-copy regions leads to the presence of a truncated *rps19* copy in IRa and a shortened *ycf1* copy in IRb [24, 34]. In this study, size variations between the *rps19* and *ycf1* copies were commonly observed. The *rps19* gene size in the LSC/IRb region was equal in every organism (279 bp), whereas the gene size of its copy in the IRa/LSC region ranged from 48 bp (in *H. dulcis* MN723868) to 240 bp (in *Z. mauritiana* and *Z. spina-christi*) (Fig. 2B). In case of *ycf1*, there was a short fragment of *ycf1* located in the IRb/SSC region with an approximate size of 1102 bp, except for *R. heterophylla*, whose case the gene size was only 300 bp (Fig. 2C). The average size of the *ycf1* large open reading frame in the SSC/IRa region was 5734 bp. The entire Ycf1 protein domain was identified in this region, although part of the domain was matched to the *ycf1* pseudogene in the IRb/SSC (Additional file 1 Fig. S3).

In addition, we identified two incidents that could be a result of assembly errors in the *R. crenata* chloroplast genome. Here, the truncated *ycf1* copy and *ycf1* appear to contradict other organisms. In this instance, inversion of IRs and SSC in the *R. crenata* chloroplast genome was observed after alignment with the other Rhamnus species (Fig. 2D). Secondly, duplicated *rps19* was completely lost from IRa. In addition, we identified a shift in the origin coordinates of the *H. acerba* MN794429 chloroplast genome (Additional file 1 Fig. S4). Here, the start coordinates were different from those of the rest of the organisms in this study. In particular, the first coordinate of the *H. acerba* MN794429 chloroplast genome began at the 5′ end of IRa, while the others began at the 5′ end of LSC.

### Re-annotation and analysis of *ycf15* and *infA* genes

Among the genes in the IRs, copies of *ycf15* were absent in 74% of the organisms in this study. In particular, *ycf15* copies were identified in seven Rhamnaceae chloroplast genomes, namely *B. wilsonii* NC_043912, *H. acerba* MN782301, *H. dulcis* MN723868, *S. parvifolium*, *V. harmandiana*, *Z. jujuba* MF781071, and *Z. jujuba* NC_030299. In case of the outgroup, there were originally two copies of *ycf15* in the *F. religiosa* chloroplast genome. The gene size of *ycf15* was 192 bp, containing no introns. Nonetheless, we were unable to predict the Ycf15 protein domain based on its protein sequence (YP_009349652). Additionally, *ycf15* copies of *C. sativa* were retrieved from *ycf15* pseudogenes of its closest species (*C. sativa* NC_026562). It appeared that the identified *ycf15* carried in-frame stop codons, which matched the fragment of the Ycf15 domain.

Multiple sequence alignment was then performed to examine the characteristics of *ycf15* among the seven Rhamnaceae species (Additional file 1 Fig. S5). First, we located a missing T-base in both copies of the *ycf15* gene of *V. harmandiana*. Interestingly, the *ycf15* gene of *B. wilsonii* NC_043912 included an intervening sequence or a 278-bp intron, whereas in the rest of the species, *ycf15* came in one piece. Moreover, the translated *ycf15* of *B. wilsonii* NC_043912 showed a longer match with the Ycf15 domain fragment and showed a sequence match from the first amino acid onwards, unlike other organisms (Additional file 1 Fig. S5). This *ycf15* annotation in Rhamnaceae appears dubious because of its ambiguous intron structure and protein domain matching.

**Fig. 3:**
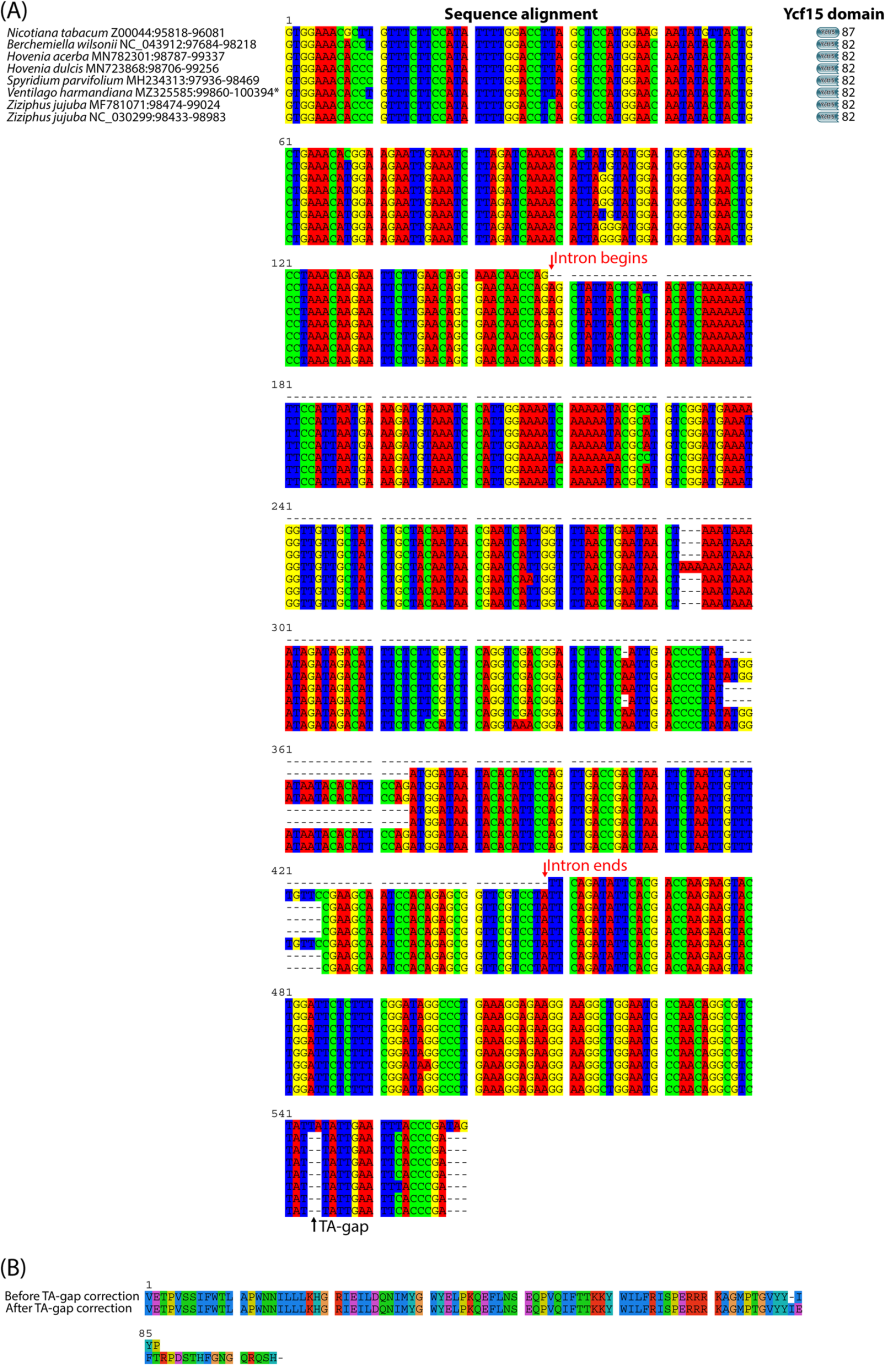
Re-annotation and analysis of the *ycf15* gene. **A** Overview of multiple sequence alignment and domain analysis of the *ycf15* gene. The alignment of the *ycf15* gene sequences from seven Rhamnaceae to the *ycf15* gene sequence of *N. tabacum* Z00044 is illustrated. The presence of an intron is indicated in red. Two missing nucleotide bases (T and A) from the *ycf15* sequences of the Rhamnaceae are pointed out by a black arrow. The predicted Ycf15 domain of each *ycf15* is illustrated on the right column. The full-length colored bar represents the presence of an entire Ycf15 domain. A truncated domain is shown by a jagged edge. The number indicates the amino acid length. Only the *ycf15* copy in IRb is shown, as its copy in IRa is identical. **B** Comparing Ycf15 amino acid sequences of *V. harmandiana* before and after TA-gap correction. A dashed line (−) indicates a stop codon

At this step, *ycf15* of *N. tabacum* Z00044 (tobacco) was chosen as a reference gene to revise the sequences of the previously identified *ycf15*. *ycf15* of *N. tabacum* is present intact, and a full-length transcript has been reported in Schmitz-Linneweber’s study [35]. Furthermore, the entire Ycf15 protein domain was detected in the protein sequence (CAA77386). Comparative sequence analysis revealed that the *ycf15* genes of *B. wilsonii* NC_043912, *H. acerba* MN782301, *H. dulcis* MN723868, *S. parvifolium*, *V. harmandiana*, *Z. jujuba* MF781071, and *Z. jujuba* NC_030299, contained intervening sequences of sizes 278, 294, 294, 277, 278, 294, and 294 bp, respectively (Fig. 3A). Moreover, a common TA-gap was observed between the sequences. Similar results were observed when the *ycf15* gene of *Barbeya oleoides* NC_040984 was re-annotated with the same reference (Additional file 1 Fig. S6). The chloroplast genome of *Barbeya oleoides* was used for genome mapping. Hence, it was skeptical whether the TA-gap was an indel mutation of Rosales lineages or an indel calling error of the genome mapping pipeline. Herein, the TA-gap in the *V. harmandiana* sequence was manually corrected, which could resolve the in-frame stop codon (Fig. 3B) and lead to the identification of the full-length Ycf15 domain. This indicated that the TA-gap was caused by an error in the genome mapping process, causing in-frame stop codons in the translated *ycf15* genes.

The *ycf15* gene of *N. tabacum* was used to re-annotate the missing *ycf15* copies in the other organisms in this study. Common characteristics, including an intervening sequence and TA-gap, existed in all Rhamnaceae chloroplast genomes (Additional file 1 Fig. S7). In both *C. sativa* and *F. religiosa* chloroplast genomes, a portion of the *ycf15* gene and TA-gap were detected (Additional file 1 Fig. S6). This part of the gene was in line with the second exon of the *ycf15* in Rhamnaceae. However, it could be interpreted as a pseudogene because of the absence of an open reading frame. Therefore, further investigation is needed to confirm the existence and structure of *ycf15* in both species.

The *infA* is a single-copy gene that has been lost in the chloroplast genomes of several species [36]. In this study, *infA* was annotated in *V. harmandiana* along with five Ziziphus species (*Z. jujuba* KX266829, *Z. jujuba* KX266830, *Z. jujuba* MW160433, *Z. mauritiana* NC_037151, and *Z. spina-christi* NC_037152), with the exception of *Z. incurva*, *Z. jujuba* MF781071, and *Z. jujuba* NC_030299. The *infA* genes of *Z. jujuba* MF781071 and *Z. jujuba* NC_030299*Z* were entirely re-annotated using the *infA* sequence of *Z. jujuba* MW160433. In contrast, the *infA* gene of *V. hamandiana* was used as a reference to study the *infA* gene of *V. leiocarpa*. However, the discovered gene contained in-frame stop codons, possibly because of an A-indel error in the *infA* sequence of *V. leiocarpa* (Additional file 1 Fig. S8).

### *V. Harmandiana* chloroplast genome features and content

**Fig. 4:**
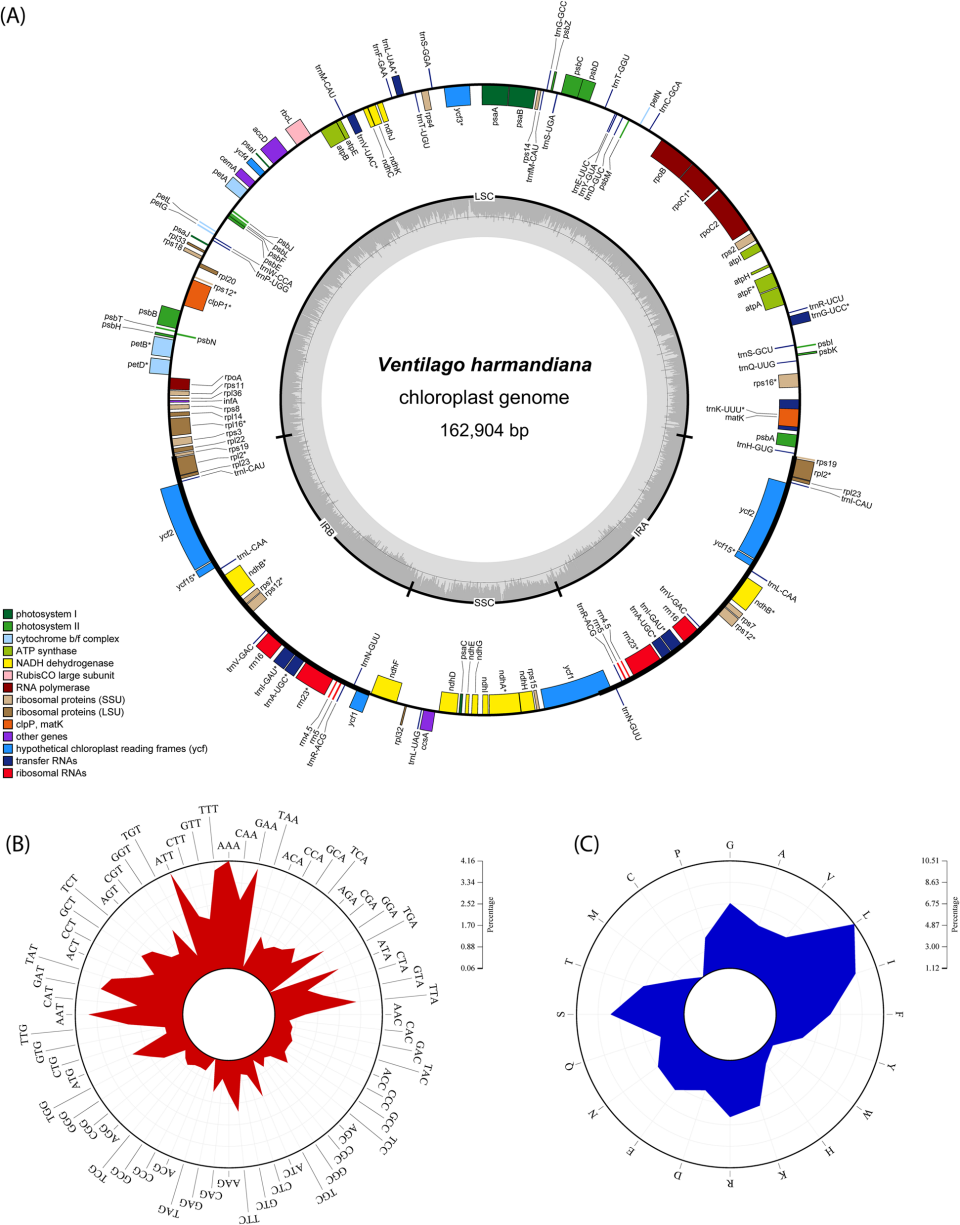
*V. harmandiana* chloroplast genome features and content. **A** Genome structure and annotation. Genes are color coded by functional categories. Genes that have introns are marked with asterisks (*). There are 89 protein-coding genes, 37 tRNA genes and 8 rRNA genes. GC content is indicated by the grey bars with the circle inside marking the 50% threshold. Thick lines indicate IR regions (IRa and IRb). **B** Codon usage and **C** amino acid usage. Circos plots illustrate the patterns of codon and amino acid usages

A newly sequenced *V. harmandiana* chloroplast genome is proposed in this study. It has a circular quadripartite structure with a size of 162,904 bp (Fig. 4A). The LSC and SSC regions separated by a pair of IRs (the size of each IR being 26,401 bp) are 90,807 and 19,295 bp long, respectively (Additional file 2 Table S2). The chloroplast genome size of *V. harmandiana* is above the average size of Rhamnaceae chloroplast genomes (161,144 bp) and is 1024 bp larger than that of its closest species, *V. leiocarpa*. The GC content of *V. harmandiana* chloroplast genome is 36.60%, which is similar to that of *H. dulcis* NC_050971 and *H. dulcis* MN723868. However, it is slightly lower than the average GC content of Rhamnaceae (36.89%).

There are 134 genes in the *V. harmandiana* chloroplast genome, including 89 protein-coding genes, 37 tRNA genes, and 8 rRNA genes (Table 1). Among these, 94 genes are single-copy and 20 genes are duplicated in the IRs (Table 1). Table 2 lists the genes and their functions. Eighteen unique genes contain one intron, and the other two genes, namely *ycf3* and *clpP1*, contain two introns. *rps12* is a trans-spliced gene, one exon of which is in the LSC, and two copies of exons are in the IR regions (Fig. 4A). The *matK* gene resides in the intron of the *trnK-UUU* gene. The tRNA genes and codon patterns of all 20 amino acids were identified (Table 3). Among the three stop codons, namely TAA, TAG, and TGA, TAA is the most abundant. Apart from stop codons, codon usage analysis identified AAA (4.16%) and TGC (0.28%) as the most and least abundant codons, respectively (Fig. 4B and Table 3). On the other hand, the most and least abundant amino acids are leucine (10.51%) and cysteine (1.12%), respectively (Fig. 4C and Table 3).

**Table 2:** List of genes in *V. harmandiana* chloroplast genome

Function	Gene list
ATP-dependent protease proteolytic subunit	clpP1*
ATP synthase	atpA, atpB, atpE, atpF*, atpH, atpI
Cytochrome b/f complex	petA, petB*, petD*, petG, petL, petN
Cytochrome complex assembly	*ccsA*
Envelope membrane protein	*cemA*
Hypothetical chloroplast reading frames	ycf1(× 2), ycf2(× 2), ycf15(× 2)*
Maturase	*matK*
NADH dehydrogenase	ndhA*, ndhB(×2)*, ndhC, ndhD, ndhE, ndhF, ndhG, ndhH, ndhI, ndhJ, ndhK
Photosystem I	psaA, psaB, psaC, psaI, psaJ, ycf3*, ycf4
Photosystem II	*psbA, psbB, psbC, psbD, psbE, psbF, psbH, psbI, psbJ, psbK, psbL, psbM, psbN, psbT, psbZ*
RubisCO large subunit	*rbcL*
Translational initiation factor	*infA*
Subunit of acetyl-CoA-carboxylase	*accD*
Large subunit of ribosomal proteins (LSU)	rpl14, rpl16*, rpl2(×2)*, rpl20, rpl22, rpl23(× 2), rpl32, rpl33, rpl36
Small subunit of ribosomal proteins (SSU)	rps2, rps3, rps4, rps7(×2), rps8, rps11, rps12(× 2, trans-spliced)*, rps14, rps15, rps16*, rps18, rps19 (× 2)
RNA polymerase	rpoA, rpoB, rpoC1*, rpoC2
Ribosomal RNAs (rRNA)	rrn4.5(×2), rrn5(× 2), rrn16(× 2), rrn23(× 2)*
Transfer RNAs (tRNA)	trnA-UGC(× 2)*, trnC-GCA, trnD-GUC, trnE-UUC, trnF-GAA, trnfM-CAU, trnG-GCC, trnG-UCC*, trnH-GUG, trnI-CAU(× 2), trnI-GAU(× 2)*, trnK-UUU*, trnL-CAA(× 2), trnL-UAA*, trnL-UAG, trnM-CAU, trnN-GUU(× 2), trnP-UGG, trnQ-UUG, trnR-ACG(× 2), trnR-UCU, trnS-GCU, trnS-GGA, trnS-UGA, trnT-GGU, trnT-UGU, trnV-GAC(× 2), trnV-UAC*, trnW-CCA, trnY-GUA

* Genes containing introns (s)

(× 2) indicates a gene with an inverted repeat

**Table 3:** The codon usage patterns of the *V. harmandiana* chloroplast genome

Amino acid	Codon	Percentage	tRNA
Phenylalanine (F)	TTT	3.828	
	TTC	2.030	*trnF-GAA*
Phenylalanine percentage		5.858	
Leucine (L)	TTA	3.184	*trnL-UAA*
	TTG	2.160	*trnL-CAA(×2)*
	CTT	2.220	
	CTC	0.719	
	CTA	1.441	*trnL-UAG*
	CTG	0.786	
Leucine percentage		10.510	
Isoleucine (I)	ATT	4.111	
	ATC	1.680	*trnI-GAU(×2)*
	ATA	2.827	*trnfM-CAU*
Isoleucine percentage		8.618	
Methionine (M)	ATG	2.279	*trnM-CAU*
Methionine percentage		2.279	
Valine (V)	GTT	2.007	*trnI-CAU(×2)*
	GTC	0.626	*trnV-GAC(×2)*
	GTA	1.989	*trnV-UAC*
	GTG	0.786	
Valine percentage		5.407	
Serine (S)	TCT	2.086	
	TCC	1.210	*trnS-GGA*
	TCA	1.505	*trnS-UGA*
	TCG	0.790	
	AGT	1.397	
	AGC	0.547	*trnS-GCU*
Serine percentage		7.534	
Proline (P)	CCT	1.531	
	CCC	0.868	
	CCA	1.136	*trnP-UGG*
	CCG	0.592	
Proline percentage		4.126	
Threonine (T)	ACT	1.989	
	ACC	0.927	*trnT- GGU*
	ACA	1.516	*trnT-UGU*
	ACG	0.618	
Threonine percentage		5.050	
Alanine (A)	GCT	2.316	
	GCC	0.871	
	GCA	1.408	*trnA-UGC(×2)*
	GCG	0.629	
Alanine percentage		5.225	
Tyrosine (Y)	TAT	2.979	
	TAC	0.771	*trnY-GUA*
Tyrosine percentage		3.750	
Histidine (H)	CAT	1.802	
	CAC	0.626	*trnH-GUG*
Histidine percentage		2.428	
Glutamine (Q)	CAA	2.685	*trnQ-UUG*
	CAG	0.771	
Glutamine percentage		3.456	
Asparagine (N)	AAT	3.657	
	AAC	1.225	*trnN-GUU(×2)*
Asparagine percentage		4.882	
Lysine (K)	AAA	4.164	*trnK-UUU*
	AAG	1.322	
Lysine percentage		5.486	
Aspartic acid (D)	GAT	3.285	
	GAC	0.782	*trnD-GUC*
Aspartic acid percentage		4.067	
Glutamic acid (E)	GAA	3.959	*trnE-UUC*
	GAG	1.311	
Glutamic acid percentage		5.270	
Cysteine (C)	TGT	0.842	
	TGC	0.276	*trnC-GCA*
Cysteine percentage		1.117	
Tryptophan (W)	TGG	1.713	*trnW-CCA*
Tryptophan percentage		1.713	
Arginine (R)	CGT	1.289	*trnR-ACG(×2)*
	CGC	0.432	
	CGA	1.389	
	CGG	0.447	
	AGA	1.870	*trnR-UCU*
	AGG	0.652	
Arginine percentage		6.078	
Glycine (G)	GGT	2.175	
	GGC	0.704	*trnG-GCC*
	GGA	2.659	*trnG-UCC*
	GGG	1.277	
Glycine percentage		6.815	
Stop codon	TAA	0.197	
Stop codon	TAG	0.071	
Stop codon	TGA	0.063	

(×2) indicates a gene with an inverted repeat

### Comparative sequence analysis unraveling the evolution of *V. harmandiana*

In total, this study included 25 chloroplast genomes from Rhamnaceae and two chloroplast genomes for the outgroup, including *C. sativa* from the family Cannabaceae and *F. religiosa* from the family Moraceae (Additional file 2 Table S2). Among the 25 chloroplast genomes of the Rhamnaceae family, eight were from the Ziziphus genus, including five from *Ziziphus jujuba*. Complete chloroplast genomes, LSC, SSC, and IR regions had average sizes of 161,144 bp, 89,136 bp, 19,109 bp, and 26,449 bp, respectively. The chloroplast genome and LSC sizes of *H. dulcis* NC_050971 were the largest, whereas those of *V. harmandiana* were the second largest. *R. heterophylla* had the smallest LSC, IR, and chloroplast genome sizes, but its SSC size was the largest. After revision of the chloroplast genomes in this study, the number of tRNA (37 genes) and rRNA (8 genes) genes was the same in all species. On an average, the total number of genes was 133. The number of genes in the *R. crenata* chloroplast genome was less than that in other organisms because the *rps19* copy in the IRa was not detectable. As the *infA* gene was re-annotated, there were more protein-coding genes (89 genes) in both Ventilago species, and all *Z. jujuba*, *Z. mauritiana*, and *Z. spina-christi* chloroplast genomes, resulting in 134 genes in these species.

**Fig. 5:**
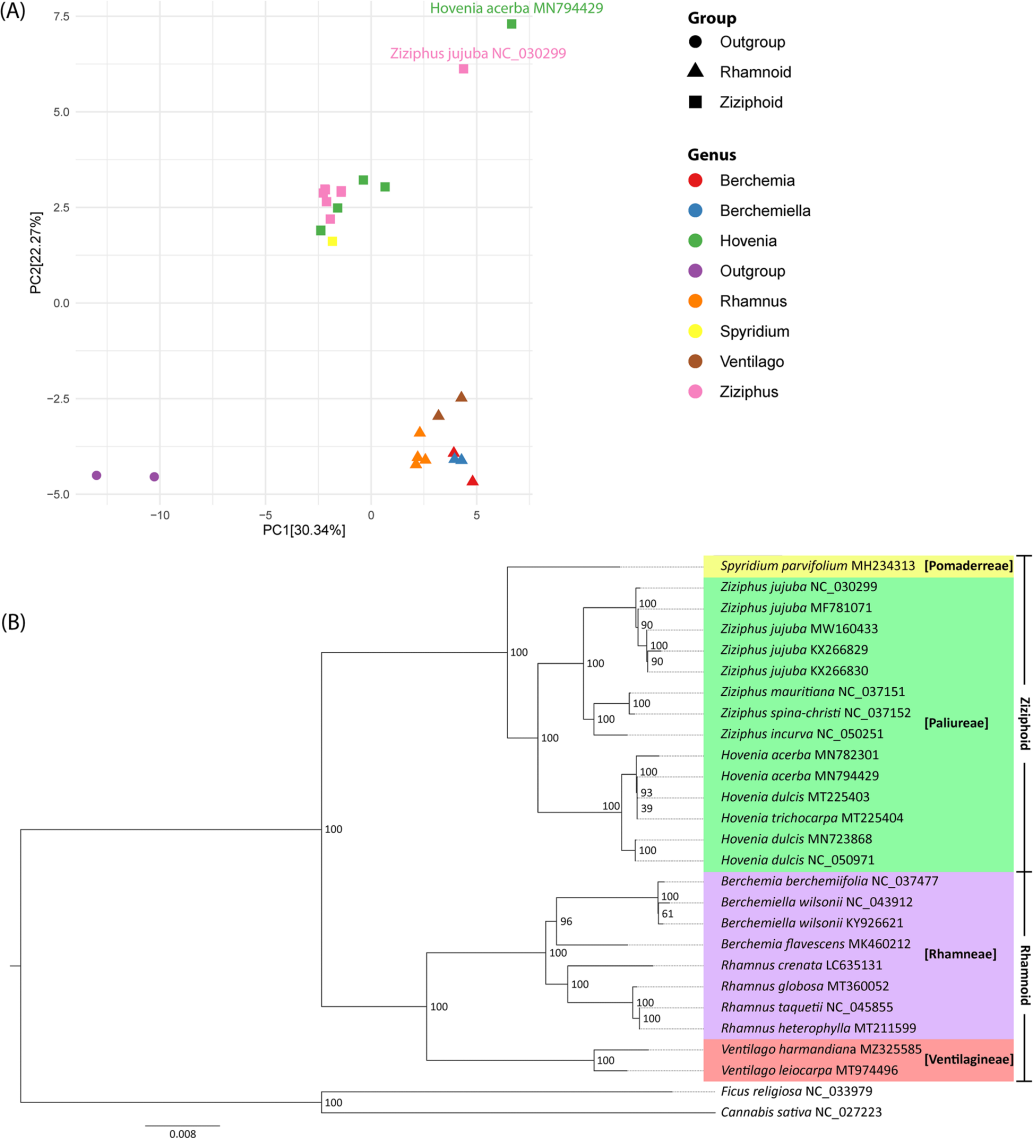
Comparative chloroplast genomes of Rhamnaceae. **A** PCA score plot of Rhamnaceae and the outgroup based on codon usage. *Z. jujuba* NC_030299 and *H. acerba* MN794429 are highlighted as they are deviated from ziziphoid group. **B** Phylogenetic tree of Rhamnaceae. Different colors correspond to tribes labelled in square brackets. The outgroup branch is not colored. Two major groups of Rhamnaceae: ziziphoid and rhamnoid are presented on the rightmost text. A number at each node indicates the maximum likelihood bootstrap value

Codon and amino acid usage analyses were performed on 86 protein-coding genes of each chloroplast genome. Both copies of *ycf15* and *infA* were excluded, as their ability to encode a functional protein remained doubtful [36, 37]. In addition, *infA* was found to be lost in several organisms. TAA (0.22% ± 0.084) was the preferred stop codon in Rhamnaceae. Apart from stop codons, AAA (4.12% ± 0.065) and TGC (0.28% ± 0.009) were the most and least abundant codons, respectively. On the other hand, the most and least abundant amino acids were leucine (10.51% ± 0.056) and cysteine (1.15% ± 0.015), respectively (Additional file 3 Table S5). PCA [38] was performed to observe codon usage patterns. Two Rhamnaceae groups, ziziphoid and rhamnoid, had distinct codon usage preferences (Fig. 5A). In particular, inter-group variation was clearly observed, while Rhamnaceae members were closely clustered within the same genus. However, *H. acerba* MN794429 and *Z. jujuba* NC_030299 were more distant from the other organisms in their genera. In this case, there were differences in gene size between *Z. jujuba* NC_030299 and the rest of *Z. jujuba*. These genes were the *atpF*, *rpl16*, *rps16*, and *rps19* copies in IRa, *ycf2*, and both copies of *ycf1*. In particular, the *atpF* coding sequence of *Z. jujuba* NC_030299 was 42 bp longer than the other *Z. jujuba*. This was due to its shorter intergenic region than that of *Z. jujuba* (Additional file 1 Fig. S9A). Similarly, the divergent structure of the *clpP* gene was observed in *H. acerba*, with a 36 bp longer coding sequence in *H. acerba* MN794429 than that in *H. acerba* MN782301 (Additional file 1 Fig. S9B).

The phylogenetic trees of 78 orthologous proteins (Additional file 1 Table S4) were reconstructed using RAxML, ML, MP, and BI methods (Fig. 5B and Additional file 1 Fig. S10A-C). Additionally, the tree reconstructed from the orthologous genes using the RAxML method was observed (Additional file 1 Fig. S10D). The *rps19* copy in the IRb and the long *ycf1* copy in the IRa were included in the analysis; only one copy was applied for the other IR genes. To the best of our knowledge, this is the highest number of orthologs as well as organisms available to reconstruct the phylogenetic tree of the family Rhamnaceae. Overall, the reconstructed trees were topologically similar, with strong bootstrap values or Bayesian posterior probability (value close to 100) supporting the majority of nodes. The Rhamnaceae chloroplast genome sequences included in this study were from two groups (ziziphoid and rhamnoid), four tribes (Paliureae, Pomaderreae, Rhamneae, and Ventilagineae), and seven genera (Berchemia, Berchemiella, Hovenia, Rhamnus, Spyridium, Ventilago, and Ziziphus). Accordingly, the phylogenetic tree contained two large groups of ziziphoid and rhamnoid (Fig. 5B). The ziziphoid group included the tribes Pomaderreae and Paliureae, whereas the rhamnoid group included the tribes Rhamneae and Ventilagineae. This clustering was congruent with a tribal classification based on molecular markers, such as *rbcL*, *trnL-F*, and ITS regions [39, 40]. In the rhamnoid clade, *V. harmandiana* and *V. leiocarpa* formed a sister clade with Berchemia, Berchemiella, and Rhamnus. Both Berchemiella species were initially grouped into *Berchemia berchemiifolia* before being grouped into another Berchemia species. In addition, we resolved the phylogenetic relationships among the three Rhamnus species. Here, *R. heterophylla* was a sister to *R. taquetii* and clustered with *R. globosa* and *R. crenata*. In the ziziphoid clade, there were two groups of Ziziphus species, in which all five *Z. jujuba* clustered together and formed a sister clade to the one containing *Z. mauritiana*, *Z. spina-christi*, and *Z. incurva*. The recent phylogeny grouped *H. acerba* MN794429, *H. dulcis* MT225403, and *H. trichocarpa* into one clade [19], which further became a sister to *H. acerba* MN782301 in our tree.

### Characteristics and comparisons of Rhamnaceae chloroplast genomes

#### Simple nucleotide repeats and long repeats

**Fig. 6:**
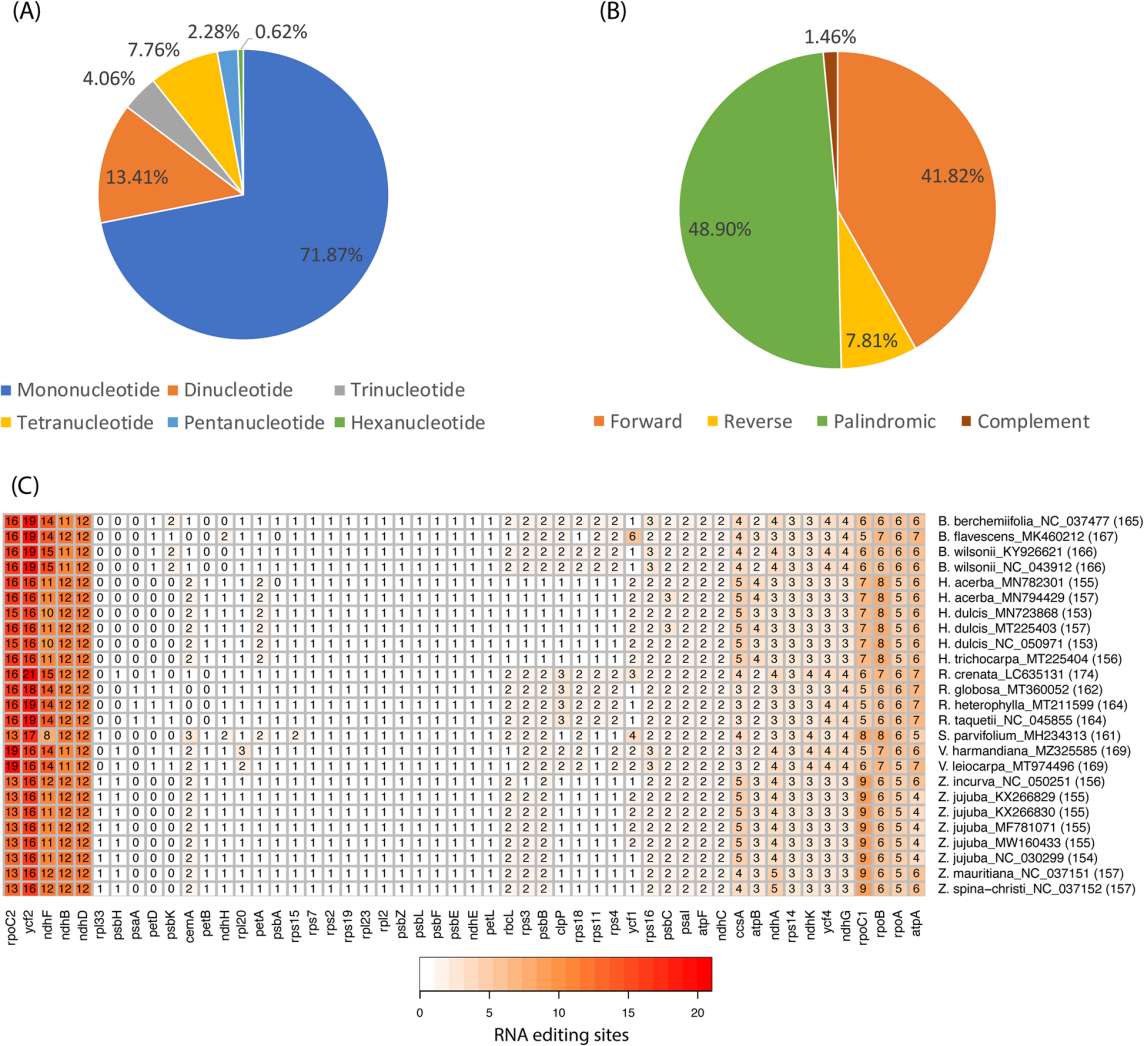
Comparisons of repeat features in Rhamnaceae chloroplast genomes. **A** Percentages of SSR types. **B** Percentages of long repeat types. **C** Comparison of RNA editing sites. The total number of RNA editing sites in each species is in the parenthesis

Among Rhamnaceae chloroplast genomes, the most abundant repeats were the mononucleotides (71.87%, Fig. 6A), and the most dominant SSR was AT-rich (Additional file 4 Table S6). Hexanucleotide repeats were found only in the genus Hovenia, *R. heterophylla*, *R. taquetii*, *S. parvifolium*, *V. leiocarpa Z. spina-christi*, and *Z. incurva*. The number of SSRs in the genus Ventilago was higher than that of the other Rhamnaceae (*V. harmandiana* = 167 SSRs and *V. leiocarpa* = 135 SSRs), while the Ziziphus and *B. flavescens* chloroplast genomes contained less than 100 SSRs (Additional file 1 Fig. S11A). Most SSRs were found in the LSC region, whereas the lowest proportion of SSRs were observed in the IRs (Additional file 1 Fig. S11B). Only 1.64% of SSRs were identified in the IR regions of *V. harmandiana*, which was less than the other Rhamnaceae. The long repeat analysis identified the palindromic repeat as a major long repeat type (48.9%) in Rhamnaceae (Fig. 6B). However, the number of the forward repeats was higher than the palindromic repeats in *V. harmandiana* and *R. heterophylla* chloroplast genomes, whereas the number of the forward and palindromic repeats in *V. leiocarpa* were equal (Additional file 1 Fig. S11C). Majorly, the long repeats located in the LSC and IR regions, and the lowest proportion was observed in the SSC (Additional file 1 Fig. S11D). However, a higher proportion of the long repeats were found in the SSC than the IRs for *R. heterophylla*. This was in relation to the finding of a very short *ycf1* gene in *R. heterophylla* in the IR regions compared to the other Rhamnaceae.

#### RNA editing sites

On average, there were 160 RNA editing sites in the Rhamnaceae chloroplast genomes. The most RNA editing sites were found in *R. crenata* (174 sites), and the lowest number of RNA editing sites were observed in *H. dulcis* MN723868 and *H. dulcis* NC_050971 (153 sites, Fig. 6C). The *rpoC2* and *ycf2* genes of both *H. acerba*, *H. dulcis* MT225403 and *H. trichocarpa* contained the same number of RNA editing sites (16 sites). The *rpoC2* gene contained the most RNA editing sites for the Ventilago (19 sites), while the most RNA editing sites were found in the *ycf2* gene for the other of the Rhamnaceae. Here, 21 RNA editing sites were observed in the *ycf2* gene of *R. crenata*. In Rhamnaceae chloroplast genomes, most of the RNA editing resulted in the conversion of serine to leucine (Additional file 5 Table S7). For the Ventilago, the second-most conversion was from alanine to valine.

#### Substitution rates and nucleotide diversity

**Fig. 7:**
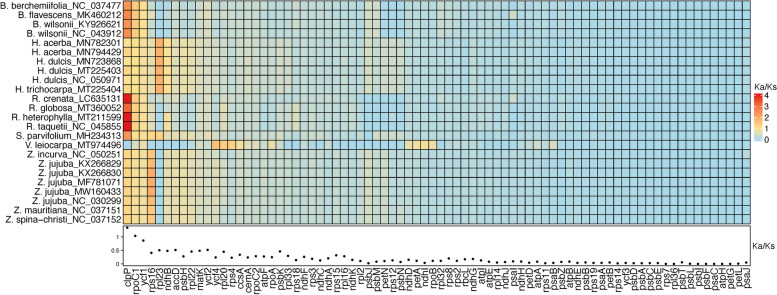
Ka/Ks ratios of protein-coding genes. The heatmap illustrated the Ka/Ks values in *V. harmandiana* compared with those in the other Rhamnaceae. The average Ka/Ks ratios were displayed by a dot plot

The Ka/Ks ratio represents selective pressure, which Ka/Ks < 1.0 for purifying, Ka/Ks = 1.0 for neutral, and Ka/Ks > 1.0 for positive selection [41]. For the Rhamnaceae chloroplast genomes, the Ka/Ks ratios of most genes were less than 1.0, meanwhile, those of the *clpP* and *rpoC1* genes were more than 1.0 (Additional file 6 Table S8), indicating both genes had undergone positive selection. Specifically, the Ka/Ks values of the *clpP* gene were higher than 1.0 by comparing *V. harmandiana* to the other Rhamnaceae, except for *V. leiocarpa* (Fig. 7 and Additional file 7 Table S9). The *rps16* and *rpl23* genes had the Ka/Ks > 1.0 in the Ventilago compared to the Ziziphus and the Hovenia, respectively (Fig. 7). The *rpoC1* and *ycf1* were less than 1.0 in the Ventilago compared to the Ziziphus (Fig. 7). The positive selection was observed in the *rps4*, *rpl20* and *ycf4* when comparing between both Ventilago. The nucleotide diversity (π) of the Rhamnaceae orthologous genes ranged from 0.002 to 0.069 with an average diversity of 0.026 (Additional file 6 Table S8). The *rpl22* and *ndhB* exhibited the highest and lowest nucleotide diversity, respectively. In particular, the π values of 31 genes (39.7% of the protein-coding genes) were higher than the average. Those genes were, for instance, the *rpl22*, *ycf1*, *clpP,* and *matK*, of which the π values were greater than 0.05. Whereas the relatively low π values (π < 0.01) were observed among the *ndhB*, *petG*, *psbJ*, *psbL*, *psbZ*, *rpl2*, *rps7*, *ycf2*, *psbJ*, *petG*, and *ycf2* genes.

## Discussion

Even though genome assembly using a combination of long and short reads achieves higher accuracy than that using long or short reads alone, additional experiments and post-processing of the draft assembly are still necessary [7, 42]. In this study, we highlight the importance of these factors in improving the accuracy of genome assembly. Using the newly sequenced chloroplast genome of *V. harmandiana* as a case study, the genome was subjected to extensive investigation and manual correction to ensure high quality and validity. Generally, the presence of a circular DNA structure and two identical IRs within chloroplast genomes is a key challenge for automated genome assemblies [43]. In our case, there were no long ONT reads covering the entire IR region. However, the absolute chloroplast structure of *V. harmandiana* was confirmed by PCR amplification of specific DNA regions, together with the number of spanning reads over the IR boundaries. Frequent errors in the ONT reads due to long homopolymer stretches and base modification [44] were polished by short reads. The remaining errors required amendment after genome assembly [7, 45, 46]. Therefore, per-base mismatches between the IRs in the *V. harmandiana* chloroplast genome were corrected using highly accurate reads from Sanger sequencing. Our findings suggest that major challenges in genome assemblies can be overcome by complementing traditional approaches.

The availability of big data-sharing resources, such as the NCBI database, offers new opportunities to conduct various studies on genomic data. Nevertheless, it is unlikely that all published genomes were entirely assembled and annotated to a high standard. This is because of the usual imperfection of computational tools, lack of standardized bioinformatics workflows, and uncorrected errors in existing genomes [14, 33, 47]. In this study, we extensively revised the annotation of the published chloroplast genomes of family Rhamnaceae, as unrevealed errors would complicate the subsequent evolutionary analysis of Rhamnaceae species. Interestingly, we identified annotation anomalies in all the published chloroplast genomes covered in this study. These included inconsistent intron splice sites of the same gene in the same species, in-frame stop codons within coding sequences, and missing genes.

Missing IR genes, including *rps19*, *ycf1*, and *ycf15*, were of particular interest. A copy of either *rps19* or *ycf1* was often misannotated. As the *rps19* and *ycf1* genes are located at the borders of the IR regions, this could possibly be a result of incomplete genome assembly. In addition, the observed size variations between the *rps19* and *ycf1* copies are considered to be a result of the contraction or expansion of the IR regions [34]. We observed high variation in the *ycf1* gene as well as the existence of a large open reading frame. Therefore, *ycf1* has been proposed as a DNA barcode for species identification [48, 49]. Moreover, a different orientation of SSC was observed in the *R. crenata* chloroplast genome. This could be either a genome assembly error or an existing form of heteroplasmy, as found in the chloroplast genomes of some plants [50]. Nonetheless, duplicated *rps19* was completely lost from the IRa of the chloroplast genome. Short-read sequencing is the main sequencing platform used in the study on *R. crenata* [26]. Furthermore, it is well known that IR assembly is particularly difficult when the IR size exceeds the read length [14]. In this case, we speculated that it was a defect in genome assembly.

Another unusual finding in this study was the *ycf15* gene. The possibility that *ycf15* is a valid protein-coding gene has not yet been proclaimed. However, it has also been annotated in several flowering plants [35, 37]. Schmitz-Linneweber et al. (2001) reported two forms of the *ycf15* gene: 1) they identified an intervening sequence of size 250–300 bp in the *ycf15* of *Arabidopsis thaliana*, *Spinacia oleracea*, *Zea mays*, and *Oenothera berteriana*, and 2) they found intact *ycf15* genes in the plastomes of *Nicotiana tabacum*, *Cuscuta reflexa*, and *Epifagus virginiana* [35]. It initially appeared that intact *ycf15* was the major gene structure among the Rhamnaceae members in this study. However, a different assumption was made based on interclade sequence matching and protein domain analysis. Moreover, the presence of an in-frame stop codon was possibly due to a TA-indel error in the second exon of *ycf15*, because the full-length Ycf15 protein domain was discovered after manual correction of the TA-indel error in *V. harmandiana*. Meanwhile, the truncated Ycf15 domain on the C-terminal side is present in other organisms. Additional evidence (such as intron information) to train gene prediction pipelines and extrinsic analysis (such as protein domain prediction) can be used to aid gene annotation. After revision of the chloroplast genomes in this study, the results showed that the numbers of protein-coding genes, tRNA genes, and rRNA genes, were similar.

The phylogeny of Rhamnaceae was then re-evaluated based on the revised chloroplast genomes. Rhamnaceae is a large plant family with a high level of morphological and genetic diversity. Previously, the classification of Rhamnaceae relied on fruit characteristics, resulting in the delimitation of two large heterogeneous tribes. The revision of tribal classification, as well as the study of taxonomic relationships between Rhamnaceae and other families (Barbeyaceae, Dirachmaceae, Elaeagnaceae, Moraceae, Rosaceae, Ulmaceae, and Urticaceae), is based on *rbcL* and *trnL-F* plastid genes combined with morphological information [15, 40]. Molecular phylogenetic analysis revealed 11 tribes [40] and three major groups of Rhamnaceae: ziziphoid, rhamnoid, and ampelozizyphoid groups [15, 16]. The majority of Rhamnaceae species belong to ziziphoid and rhamnoid groups [39]. Although *rbcL* and *trnL-F* sequence data have been used for intrafamilial and suprageneric phylogenetic studies [15], more genetic information is needed for a better resolution of Rhamnaceae phylogeny. In particular, the combination of the *trnL-F* region of chloroplast DNA and the nuclear ribosomal DNA internal transcribed spacer (ITS) regions has been used to study Rhamnaceae tribes and genera [39, 51, 52]. These studies largely support the taxonomic study by Richardson et al. [40] and successfully validate controversial genera such as Rhamnus and Frangula. However, the phylogenetic relationships of polyphyletic genera (for example, Ziziphus) and genera without assigned tribes, remain unresolved by using only a few marker genes. Moreover, incongruence between genes can be observed by using too few genes, which makes phylogenetic reconstruction more error prone [53]. More genetic data are therefore urgently needed.

The phylogenetic trees of Rhamnaceae have been built based on whole chloroplast genomes. However, the use of highly conserved and varied loci in phylogenetic studies requires consideration. Highly conserved genetic information usually resolves higher levels of phylogenetic trees (for example, family and genus) than highly variable genetic information. Conversely, the inclusion of exceptional insertions or deletions in the phylogenetic analysis could result in bias clustering towards a specific taxon. Therefore, orthologous identification was performed prior to phylogenetic reconstruction in this study. The phylogenetic tree at low taxonomic levels of Rhamnaceae was reconstructed using 78 orthologous protein-coding genes from 25 Rhamnaceae members and two organisms for the outgroup. As a greater number of Rhamnaceae species were included in the analysis, our phylogenetic tree added up the coverage of phylogenetic relationships between Rhamnaceae species to that of other studies [20, 24, 54, 55]. Furthermore, the clusters of Rhamnaceae organisms based on codon usage were in line with the molecular phylogenetic tree. Minor deviations of *Z. jujuba* NC_030299 and *H. acerba* MN794429 from their ziziphoid groups were observed based on codon usage. This could be a result of the imprecise prediction of intron-splice sites. Further investigation is needed to resolve the unaligned genes in both the *Z. jujuba* and *H. acerba* chloroplast genomes.

Additionally, the SSRs were reported as informative DNA markers to differentiate organisms at the lower taxonomic levels because of the high length variation and polymorphism [56, 57]. These regions were successfully used for the analysis of intraspecific genetic variation in the Ziziphus [24] and ginsengs [58]. Consistently, we found the proportion of SSRs in each chloroplast region was different between species, but it was fairly similar within the same species, such as *B. wilsonii*, *H. acerba* and *Z. jujuba*. Moreover, because short polyadenine (polyA) or polythymine (polyT) repeats are the main component of SSRs in chloroplast genomes [59], the most dominant A/T motifs may contribute to overall high AT content of the Rhamnaceae chloroplast genomes. RNA editing is the modification of the transcripts that is observed in viruses and various eukaryotes including plants [60]. Our study found that most of the RNA editing sites led to the conversion of serine to leucine, which is in accordance with the general characteristic of RNA editing in higher plants [61, 62]. Besides, the number of predicted editing sites was similar among closely related species. The analysis of nucleotide substitution of the orthologous genes reflected the high conservation of the chloroplast genomes, as most genes were strongly subjected to purifying selection in the evolutionary process. However, the *clpP* gene was strongly subjected to positive selection, and had relatively high nucleotide diversity. This could infer that the gene could be important in the adaptive evolution of the Rhamnaceae. The positive selection of the *clpP* in several plant lineages was found to be involved in repeated duplication [63].

Our study shows that genomic information at both the DNA and protein levels could support a tribal classification of Rhamnaceae and could clarify the phylogeny at low taxonomic levels. The completeness of the genomes is a basic requirement for subsequent comparative genomic analyses. In this study, we also demonstrate that extensive corrections of genome sequences and revision of annotations are essential, as errors in existing DNA sequence data could propagate to newly sequenced genomes and lead to endless flaws in consecutive genomic analyses.

## Conclusions

In this study, we made use of the key advantages of different DNA sequencing platforms, to congregate the entire chloroplast genome of *V. harmandiana* and to ensure its high quality. In particular, ONT long reads were used to generate a draft assembly, and Illumina HiSeq short reads were integrated to polish the genome. Moreover, we demonstrated that a combination of PCR amplification, investigation of coverage depth, and Sanger sequencing for nucleotide base corrections could lead to elucidation of the complete chloroplast genome, even if the read length was shorter than the IR size. We then examined the features and content of the chloroplast genome.

Major gene functions of the *V. harmandiana* chloroplast genome, such as photosynthesis and genome replication, were as described in other plants. The reported chloroplast genome is not only a useful resource, but will also aid in the genetic exploration of this medicinal plant, as well as in the study of Rhamnaceae phylogeny. In this regard, we report the most complete phylogenetic tree of Rhamnaceae based on chloroplast genome information. It included a higher number of Rhamnaceae species than that in the dispersed phylogenetic relationships elucidated in other studies. However, during genome revision, we identified putative assembly errors and annotation anomalies in published chloroplast genomes of this family. This indicates the importance of revising the genome annotation prior to genomic analysis. Moreover, reviewing the completeness and correctness of the annotation before genome submission is superior. This is necessary to avoid the propagation of errors from reference genomes to newly assembled genomes, which could be carried on to downstream analysis and biological interpretation.

## Methods

### Plant material and sampling

Sample collection of *V. harmandiana* was permitted by the private land owner in Trang province, Thailand, Mr. Wanlop Pengphan. The collection of the *V. harmandiana* covering various plant parts for research and related purposes was done with the permission and supervision of the local authority. Plant samples were collected as previously described [29]. Briefly, *V. harmandiana* leaves were collected in March 2019 in Trang Province, Thailand (lat. 7°47′12.8″ N, long. 99°30′55.0″ E; altitude 104 m a.s.l.). The collected samples were identified by the author, Dr. Narong Nuntasaen from Department of National Parks, Wildlife and Plant Conservation, Ministry of Natural Resources and Environment. All samples with the voucher specimen accession code, BKF no. 35203, were deposited at the Forest Herbarium, Department of National Parks, Wildlife and Plant Conservation in Bangkok, Thailand. The samples were immediately washed with tap water to remove dirt and kept on dry ice during transport to the laboratory. The samples were stored at − 80 °C until use.

### DNA extraction

The leaves were soaked in 0.05% Tween 20 to remove fungal spores and bacterial cells from their surfaces, and washed several times with distilled water. To extract DNA, 1 g of young leaves was ground to a fine powder using liquid nitrogen. Then, 7.5 ml of freshly prepared CTAB buffer (20 mM EDTA pH 8.0, 100 mM Tris-HCl pH 8.0, 1.5 M NaCl, 2% CTAB, 1% β-mercaptoethanol, 2% polyvinylpyrrolidone M.W. 10,000) was added, and incubated at 60 °C for 2 h with intermittent shaking every 10 min. The samples were centrifuged at 15,000 rpm for 30 min at room temperature and the aqueous phase was transferred to a new tube. Next, 1 V of phenol-chloroform-isoamyl alcohol (25:24:1) was added, inverted gently for 1 min, and centrifuged at 15,000 rpm for 15 min. This step was repeated using chloroform until the upper phase was translucent and no interphase protein appeared. The aqueous phase (upper phase) was transferred into a new tube without chloroform (lower phase). To precipitate the DNA, 2 V of isopropanol and 1/3 V of 3 M sodium acetate (pH 5.2) were added. The sample was then kept at − 20 °C for 1 h, followed by centrifugation at 15,000 rpm for 15 min. The DNA pellet was washed twice with 70% ethanol and absolute ethanol, and resuspended in TE buffer. DNA samples were supplemented with DNase-free RNase A (20 μg/ml) and incubated for 30 min at 37 °C to remove RNA. Finally, the DNA was re-purified using QIAGEN Genomic-tip 100/G (cat no.13343) according to the manufacturer’s protocol to obtain high-quality DNA (OD 260/280 and 260/230 of 2.0).

### Chloroplast genome sequencing

*MinION sequencing:* To obtain the chloroplast sequencing reads, two library kits were used for DNA library preparation: the Rapid Barcoding Sequencing kit (SQK-RAD004, ONT, UK) and the 1D Genomic DNA sequencing kit (SQK-LSK109, ONT). A total of 500 ng of DNA was used for SQK-RAD004 input and 1500 ng was used for SQK-LSK109 input. We followed the protocol recommended by ONT, except for the DNA shearing step. Each DNA library was loaded onto an R9.4/FLO-MIN106 flow cell (ONT) on a MinION Mk1B for 48 h. Base-calling was performed using the local-based software GUPPY version v2.1.3 (ONT).

*PromethION sequencing:* To obtain the highest sequencing yield of long reads, the PromethION platform was selected for gDNA sequencing. The DNA library was prepared using SQK-LSK109 (ONT). A total of 2500 ng of DNA was used as input. The DNA library was loaded onto a PromethION flow cell (ONT) and incubated for 48 h. Base-calling was performed using the local-based software GUPPY version v2.1.3 (ONT).

A short-read high-throughput Illumina platform (HiSeq, Illumina, Inc., USA) was used to improve the accuracy of the final nanopore sequence. A TruSeq DNA polymerase chain reaction (PCR)-free library was prepared and 100-bp paired-end reads were generated.

The long- and short-read raw data of the chloroplast has been deposited in the Sequence Read Archive (SRA) repository, https://www.ncbi.nlm.nih.gov/sra/PRJNA906747.

### Chloroplast genome assembly

Hybrid assemblies of ONT (combined reads from *MinION* and *PromethION* sequencing) and Illumina data were performed using default settings for circular chloroplast genomes. The ONT adapters in Nanopore reads were trimmed using Porechop v0.2.3 (https://github.com/rrwick/Porechop). The whole genome Nanopore reads with a mean quality score of 9 and a length of 1000 bases were retained from QC step using NanoFilt v2.5.0 [64]. The high-quality reads were then mapped to the closely related species chloroplast genome of *Barbeya oleoides* NC_040984.1, to identify chloroplast reads using Minimap2 [65]. To reduce complexity, all mapped reads were subjected to de novo assembly using Unicycler v0.4.7 [66]. Six rounds of Pilon v1.23 [67] polishing with Illumina data were applied iteratively on the circular draft chloroplast genome.

### Validation of chloroplast inverted repeat regions

PCR amplification of DNA regions spanning over the four IR boundaries of the circular draft chloroplast genome was performed to determine the quadripartite structure of the *V. harmandiana* chloroplast genome (Fig. 1C and Additional file 1 Fig. S12). Primer sets were designed using the assembled sequences. Four pairs of PCR primers were designed to cover the LSC-IRb junction, IRb-SSC junction, SSC-IRa junction, and IRa-LSC junction (primers VharA–D, Additional file 2 Table S1 and Fig. 1B). Specific primers for the *18S rDNA* gene (18S_FA: AAC CTG GTT CCT GCC AG and 18S_RB: TGA TCC TTC TGC AGG TTC AC) were used as positive controls [68]. Five PCR reactions were performed, including reactions for the four boundaries and the positive control. Each PCR reaction mixture consisted of 0.2 μM of each primer, 200 μM of each dNTP, 1X of ThermoPol® Reaction Buffer (New England Biolabs, USA), 2.5 U of *Taq* DNA polymerase (New England Biolabs, USA), and 15 ng of the chloroplast DNA. PCR reaction mixtures were adjusted to a final volume of 50 μL using sterile nuclease-free water. The PCR conditions were as follows: initial denaturation at 95 °C for 5 min; 30 cycles of 95 °C for 5 min, 56 °C for 30 s, and 68 °C for 2 min; and final extension at 68 °C for 10 min. The amplicons were analyzed using 1.5% agarose gel electrophoresis with FluoroVue™ Nucleic Acid Gel Stain (SmoBio, Taiwan) and documented using G:BOX Chemi XRQ gel documentation (Syngene™, USA).

Local sequence alignment was conducted between IRb and reverse-complemented IRa (revIRa) to identify mismatched nucleotide bases between the IRs, and the correct bases were determined by Sanger sequencing. Fourteen primer pairs were designed to cover all 25 mismatched regions within the two IRs (primers VharIR1–14; Additional file 2 Table S1). Fourteen PCRs were performed using the same reaction components as described previously. The PCR conditions were as follows: initial denaturation at 95 °C for 5 min; 30 cycles of 95 °C for 5 min, 55 °C for 1 min, and 68 °C for 2 min; and final extension at 68 °C for 10 min. The PCR amplicons were purified using NucleoSpin Gel and PCR Clean-up (Macherey-Nagel, Germany), according to the manufacturer’s instructions. The purified amplicons were verified using gel electrophoresis and a NanoDrop spectrophotometer (Thermo Fisher, USA) before being sent for sequencing (Macrogen Inc., Republic of Korea). Nucleotide sequences were trimmed and manually edited using electropherograms. The edited sequences of each amplicon were aligned to the IRb and revIRa using Seaview version 5.0.5 [69], and the nucleotide sequences of both IRb and revIRa were manually corrected.

### Genome annotation

The GeSeq online tool [12] was used for annotation of the *V. harmandiana* chloroplast genome, and the OrganellarGenomeDraw (OGDRAW) tool [70] was used to draw the circular chloroplast gene map. The complete chloroplast genome of *V. harmandianad* has been deposited in the NCBI repository, https://www.ncbi.nlm.nih.gov/nuccore/MZ325585.

The complete chloroplast genomes of 24 Rhamnaceae members and two chloroplast genomes of *Cannabis sativa* cultivar Yoruba Nigeria and *Ficus religiosa* voucher Ronsted86, were retrieved from the NCBI database (Additional file 2 Table S2). *C. sativa* and *F. religiosa* were the outgroup in the phylogenetic analysis. The obtained sequence data, together with the *V. harmandiana* chloroplast sequence, were revised prior to further analysis. For the revision of published chloroplast genomes, the Muscle program [71] implemented in the SeaView software version 5.0.5 [69] was used for multiple sequence alignment. Database searches were performed using the BLAST algorithm [72] within the NCBI database. Protein domain prediction was performed using the hmmscan program [73] in HMMER webserver version 2.41.2 [74].

### Codon and amino acid usage analysis

The codon usage and amino acid preference of individual chloroplast genomes were calculated based on coding sequences using the CMG biotool [75]. The results are illustrated in radar plots. Principle component analysis (PCA) was performed on the codon usage matrix to compare codon usage across different chloroplast genomes by using R programming language. The results were summarized in a two-dimensional plot of the first two principal components.

### Phylogenetic analysis of orthologs

Seventy-eight orthologous proteins (Additional file 2 Table S4) were identified from 25 Rhamnaceae members and the outgroup, and were used in phylogenetic reconstruction. Amino acid sequence multiple alignments of individual orthologs were performed using the Muscle software version 5.1 [76]. The aligned sequences were then trimmed and concatenated using trimAl version 1.2 [77]. Phylogenetic analysis was performed using the randomized axelerated maximum likelihood (RAxML) method under raxmlGUI software environment version 2.0 [78], the maximum likelihood (ML), and the maximum parsimony (MP) method using MEGA software version 11 [79]. Bootstrapping was performed 500 times to obtain the bootstrap confidence value of the tree branches. For Bayesian inference (BI) method, the best-fit model, JTT + F + I + G4 model, was computed by ModelFinder [80] in IQ-TREE version 2.2 [81]. BI was performed on MrBayes version 3.2 with two independent Markov chain Monte Carlo (MCMC) runs. Each of four MCMC chains was run for 1000,000 generations and the first 25% of samples from the beginning of the chain were discarded as burn-in. All reconstructed trees were visualized with FIGTREE version 1.4.4 (http://tree.bio.ed.ac.uk/software/figtree/).

### Comparative analysis of chloroplast genome features

Repeat structure analysis was performed to identify both simple sequence repeats (SSRs) and long repeats. The SSRs or microsatellites with motif sizes of one to six nucleotides were examined by MicroSAtellite (MISA) [82]. The minimum number of repeats was defined as 10 for mononucleotides, five for dinucleotides, four for tri-nucleotides and three for tetra-, penta-, and hexa-nucleotides. The long repeat sequences were detected by REPuter program [83]. The minimum repeat size was 30 bp, the maximum computed repeats was 90 bp and Hamming distance equal was set to three. The match directions for the long repeats include forward or direct, reverse, palindromic and complement matches.

RNA editing sites of protein-coding genes were predicted using the online Plant RNA Editing Prediction and Analysis Computer (PREPACT 3.0) [84, 85]. The prediction was performed on the BLASTX analysis mode using *Cucumis sativus* NC_007144 as a reference sequence.

Protein-coding sequences of individual orthologs were aligned by the online MUSCLE software (https://www.ebi.ac.uk/Tools/msa/muscle/) [86] prior to calculation of the synonymous (Ks) and non-synonymous (Ka) substitution rates and nucleotide diversity with DnaSP version 6.12.03 [87]. The average Ka/Ks ratio of each gene was calculated to obtain the selection patterns of Rhamnaceae. Besides, the Ka/Ks values in *V. harmandiana* compared with those in the other Rhamnaceae were observed.

## Supplementary Information

**Additional file 1: Figure S1.** The number of spanning reads over the IR boundaries. (A) Boundary A locates between LSC and IRb. (B) Boundary B locates between IRb and SSC. (C) Boundary C locates between SSC and IRa. (D) Boundary D locates between IRa and LSC. The number of ONT reads spanning over the four IR boundaries at different distances (nt) from the boundaries are illustrated as bar charts. Large single-copy (LSC); inverted repeats (IRs) and small single-copy (SSC). **Figure S2.** Comparative gene arrangement in IR regions of Rhamnaceae, *C. sativa* and *F. religios*. Each box represents a gene. The *rps19* and *ycf1* genes (bold and underline) are located at the borders of IR regions. A missing gene that was re-annotated is highlighted in red. A gene in LSC is represented by an orange box. A gene in IR is in a blue box and a gene in both LSC and IR regions is in a white box. The *C. sativa* NC_027223 and *F. religiosa* NC_033979 are the outgroup of phylogenetic analysis. Reversed inverted repeat (revIR). **Figure S3.** Domain analysis of Ycf1 protein sequences. The full-length colored bar represents the presence of an entire Ycf1 domain. A shorter colored bar within the grey bar indicates the matching part of the domain. A truncated domain is shown by a jagged edge. The number indicates the amino acid length. *Ycf1* copy in IRb (ycf1_IRb) and *ycf1* gene in IRa (ycf1_IRa). **Figure S4.** A shift in the origin of *H. acerba* MN794429 chloroplast genome and correction. Suggested origin is presented in red color. **Figure S5.** Multiple alignment of the *ycf15* gene sequences and matching domain fragments. This figure shows the alignment of the deposited *ycf15* DNA sequences from six Rhamnaceae organisms and the *ycf15* sequence of *V. harmandiana*. The predicted Ycf15 domain of each *ycf15* is illustrated on the right column. A missing T-base from the *ycf15* sequence of *V. harmandiana* is pointed out by an arrow. The full-length colored bar represents the presence of an entire Ycf15 domain. A truncated omain is shown by a jagged edge. The number indicates the amino acid length. Only the *ycf15* copy in IRb is shown, as its copy in IRa is identical. **Figure S6.** Re-annotation of the *ycf15* gene in *Barbeya oleoides* NC_040984 and the outgroup. The alignment of the *ycf15* gene sequences of *Barbeya oleoides*, *C. sativa* and *F. religiosa* to the *ycf15* gene sequence of *N. tabacum* Z00044 is illustrated. The TA-gap is pointed out by a black arrow. The predicted Ycf15 domain of each *ycf15* is illustrated on the right column. The full-length colored bar represents the presence of an entire Ycf15 domain. A truncated domain is shown by a jagged edge. The number indicates the amino acid length. Only the *ycf15* copy in IRb is shown, as its copy in IRa is identical. **Figure S7.** Re-annotation of the *ycf15* gene in Rhamnaceae. (A) The alignment of the reference gene sequence to the first exon and (B) to the second exon of the *ycf15* gene in Rhamnaceae organisms. The figure shows the multiple sequence alignment using *N. tabacum* Z00044 as a reference to re-annotate the *ycf15* gene of the organisms in this study. TA-gaps in the *ycf15* sequences of the Rhamnaceae are pointed out by a black arrow. The predicted Ycf15 domain of each *ycf15* is illustrated on the right column. The full-length colored bar represents the presence of an entire Ycf15 domain. A truncated domain is shown by a jagged edge. The number indicates the amino acid length. Only the *ycf15* copy in IRb is shown, as its copy in IRa is identical **Figure S8.** Re-annotation of *infA* gene in *V. leiocarpa*. (A) The alignment of *infA* DNA sequences between *V. harmandiana* and *V. leiocarpa*. A-gap is pointed out by an arrow. (B) Comparing amino acid sequences of the *infA* between *V. harmandiana* and *V. leiocarpa*. A dashed line (−) indicates a stop codon. **Figure S9.** Comparisons of the *atpF* and *clpP* gene structures. (A) Comparing the *atpF* gene structure of *Z. jujuba* species. (B) Comparing the *clpP* gene structure of *H. acerba* species. A blue box represents a coding region and the red wavy line represents an intergenic region. The base position of the *atpF* in *Z. jujuba* NC_030299 and the *clpP* in *H. acerba* MN794429 is used as a reference for each gene. **Figure S10.** Phylogenetic trees of Rhamnaceae reconstructed from the orthologous proteins (A) using ML method, (B) using MP method, (C) using BI method and (D) the tree reconstructed from the orthologous genes using RAxML method. Different colors correspond to tribes labelled in square brackets. The outgroup branch is not colored. Two major groups of Rhamnaceae: ziziphoid and rhamnoid are presented on the rightmost text. A number in each tree indicates the ML bootstrap value, the MP bootstrap value, the posterior probability of BI, the ML bootstrap value, respectively. **Figure S11.** Characteristics and comparisons of Rhamnaceae chloroplast genome features. (A) Comparison of the SSR types. (B) Distribution of SSRs in LSC, SSC and IR regions. (C) Comparison of the long repeat types. (D) Distribution of long repeats in LSC, SSC and IR regions. The total number of SSRs and long repeat sequences in each species is in the parenthesis. **Figure S12.** The original image of gel in Fig. 1C before cropping and inverting color.

**Additional file 2: Table S1.** List of primers. **Table S2**. List of 25 Rhamnaceae complete chloroplast genomes and the outgroup for the phylogenetic analysis. **Table S3.** List of re-annotated genes. **Table S4.** List of orthologous genes for phylogenetic reconstruction.

**Additional file 3: Table S5.** Codon usage of 86 protein-coding genes of each chloroplast genome.

**Additional file 4: Table S6.** SSRs of 25 Rhamnaceae chloroplast genomes.

**Additional file 5: Table S7.** Changes of amino acids from RNA editing

**Additional file 6: Table S8.** The Ka/Ks ratio values and nucleotide diversity of protein-coding genes of the Rhamnaceae

**Additional file 7: Table S9.** The Ka/Ks ratio comparisons with *V. harmandiana*

## Data Availability

The complete chloroplast genome of *V. harmandiana*d has been deposited in the NCBI repository, https://www.ncbi.nlm.nih.gov/nuccore/MZ325585. The original contributions presented in the study can be found in the article and additional files, and further inquiries can be directed to the corresponding authors.
