# Supplementary material for: Revisiting chloroplast genomic landscape and annotation towards comparative chloroplast genomes of Rhamnaceae
Source: BMC Plant Biol. 2023 Jan 28;23:59. doi: 10.1186/s12870-023-04074-5 (PMC9883906; doi:10.1186/s12870-023-04074-5)
Supplement: Supplementary file 1 — Additional file 1: Figure S1. The number of spanning reads over the IR boundaries. (A) Boundary A locates between LSC and IRb. (B) Boundary B locates between IRb and SSC. (C) Boundary C locates between SSC and IRa. (D) Boundary D locates between IRa and LSC. The number of ONT reads spanning over the four IR boundaries at different distances (nt) from the boundaries are illustrated as bar charts. Large single-copy (LSC); inverted repeats (IRs) and small single-copy (SSC). Figure S2. Comparative gene arrangement in IR regions of Rhamnaceae, C. sativa and F. religios. Each box represents a gene. The rps19 and ycf1 genes (bold and underline) are located at the borders of IR regions. A missing gene that was re-annotated is highlighted in red. A gene in LSC is represented by an orange box. A gene in IR is in a blue box and a gene in both LSC and IR regions is in a white box. The C. sativa NC_027223 and F. religiosa NC_033979 are the outgroup of phylogenetic analysis. Reversed inverted repeat (revIR). Figure S3. Domain analysis of Ycf1 protein sequences. The full-length colored bar represents the presence of an entire Ycf1 domain. A shorter colored bar within the grey bar indicates the matching part of the domain. A truncated domain is shown by a jagged edge. The number indicates the amino acid length. Ycf1 copy in IRb (ycf1_IRb) and ycf1 gene in IRa (ycf1_IRa). Figure S4. A shift in the origin of H. acerba MN794429 chloroplast genome and correction. Suggested origin is presented in red color. Figure S5. Multiple alignment of the ycf15 gene sequences and matching domain fragments. This figure shows the alignment of the deposited ycf15 DNA sequences from six Rhamnaceae organisms and the ycf15 sequence of V. harmandiana. The predicted Ycf15 domain of each ycf15 is illustrated on the right column. A missing T-base from the ycf15 sequence of V. harmandiana is pointed out by an arrow. The full-length colored bar represents the presence of an entire Ycf15 domain. A truncated oma [file 12870_2023_4074_MOESM1_ESM.pdf]

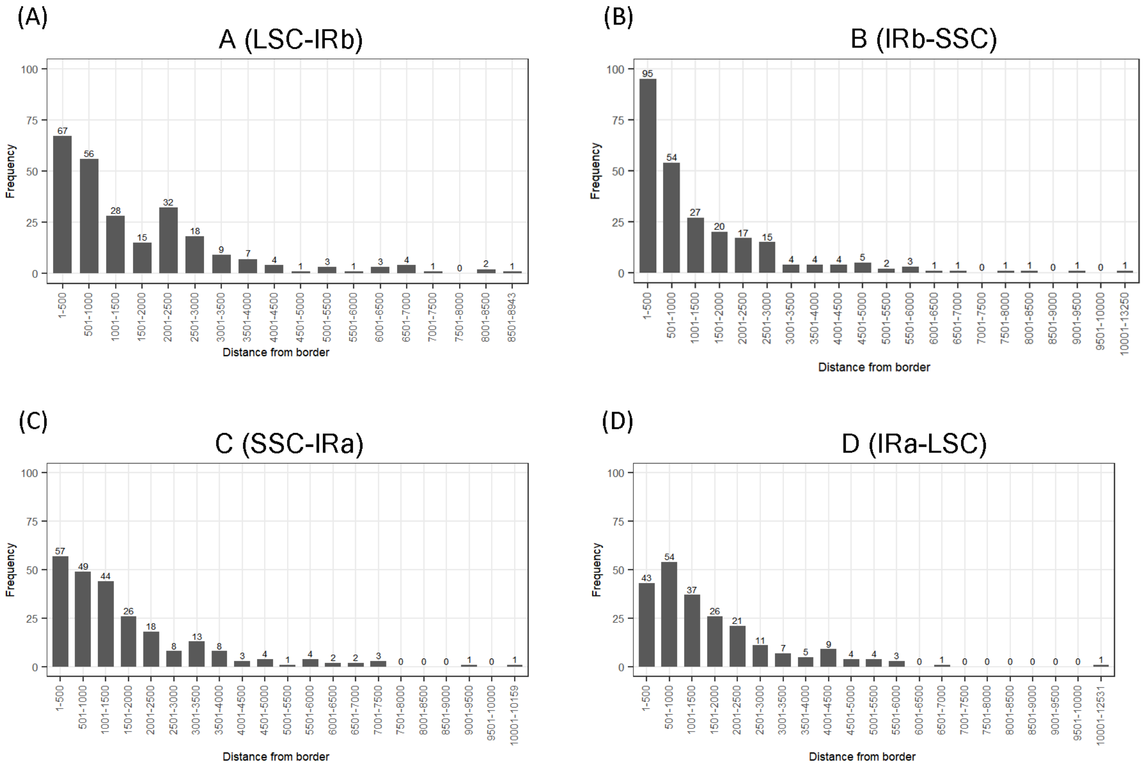

Figure S1. The number of spanning reads over the IR boundaries. (A) Boundary A locates between LSC and IRb. (B) Boundary B locates between IRb and SSC. (C) Boundary C locates between SSC and IRa. (D) Boundary D locates between IRa and LSC. The number of ONT reads spanning over the four IR boundaries at different distances (nt) from the boundaries are illustrated as bar charts. Large single-copy (LSC); inverted repeats (IRs) and small single-copy (SSC).

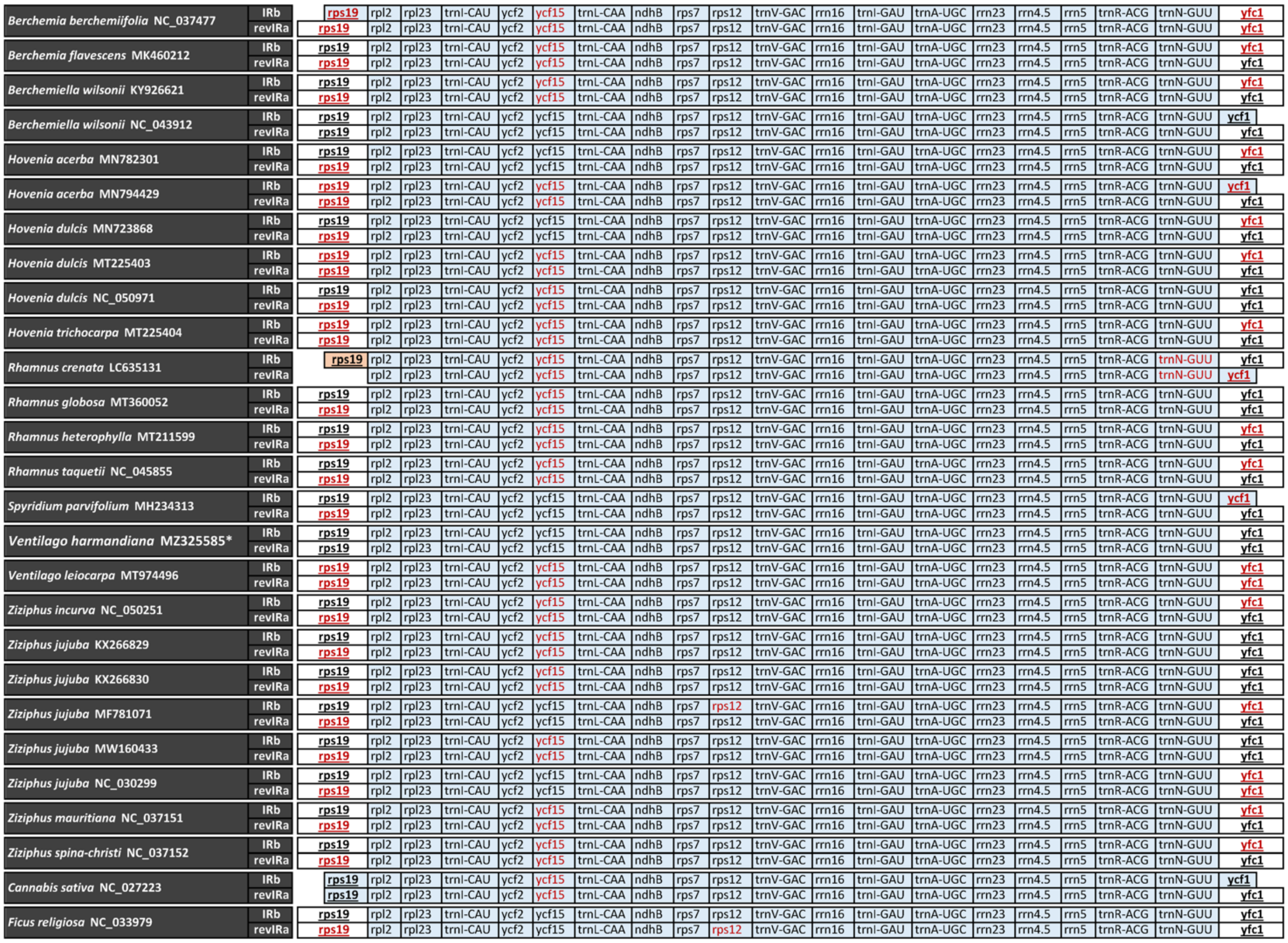

Figure S2. Comparative gene arrangement in IR regions of Rhamnaceae, *C. sativa* and *F. religios*. Each box represents a gene. The *rps19* and *ycf1* genes (bold and underline) are located at the borders of IR regions. A missing gene that was re-annotated is highlighted in red. A gene in LSC is represented by an orange box. A gene in IR is in a blue box and a gene in both LSC and IR regions is in a white box. The *C. sativa* NC_027223 and *F. religiosa* NC_033979 are the outgroup of phylogenetic analysis. Reversed inverted repeat (revIR).

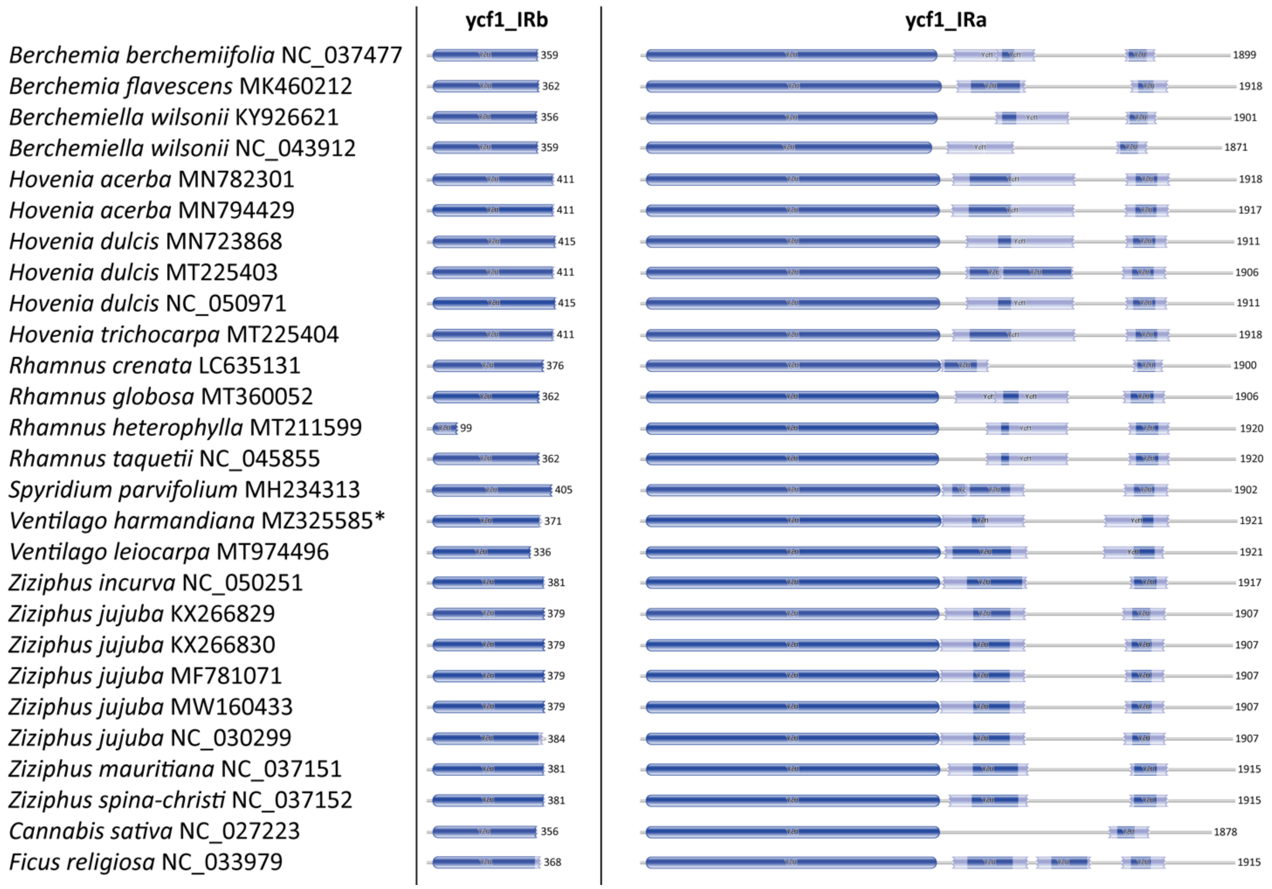

Figure S3. Domain analysis of Ycf1 protein sequences. The full-length colored bar represents the presence of an entire Ycf1 domain. A shorter colored bar within the grey bar indicates the matching part of the domain. A truncated domain is shown by a jagged edge. The number indicates the amino acid length. *Ycf1* copy in IRb (ycf1_IRb) and *ycf1* gene in IRa (ycf1_IRa).

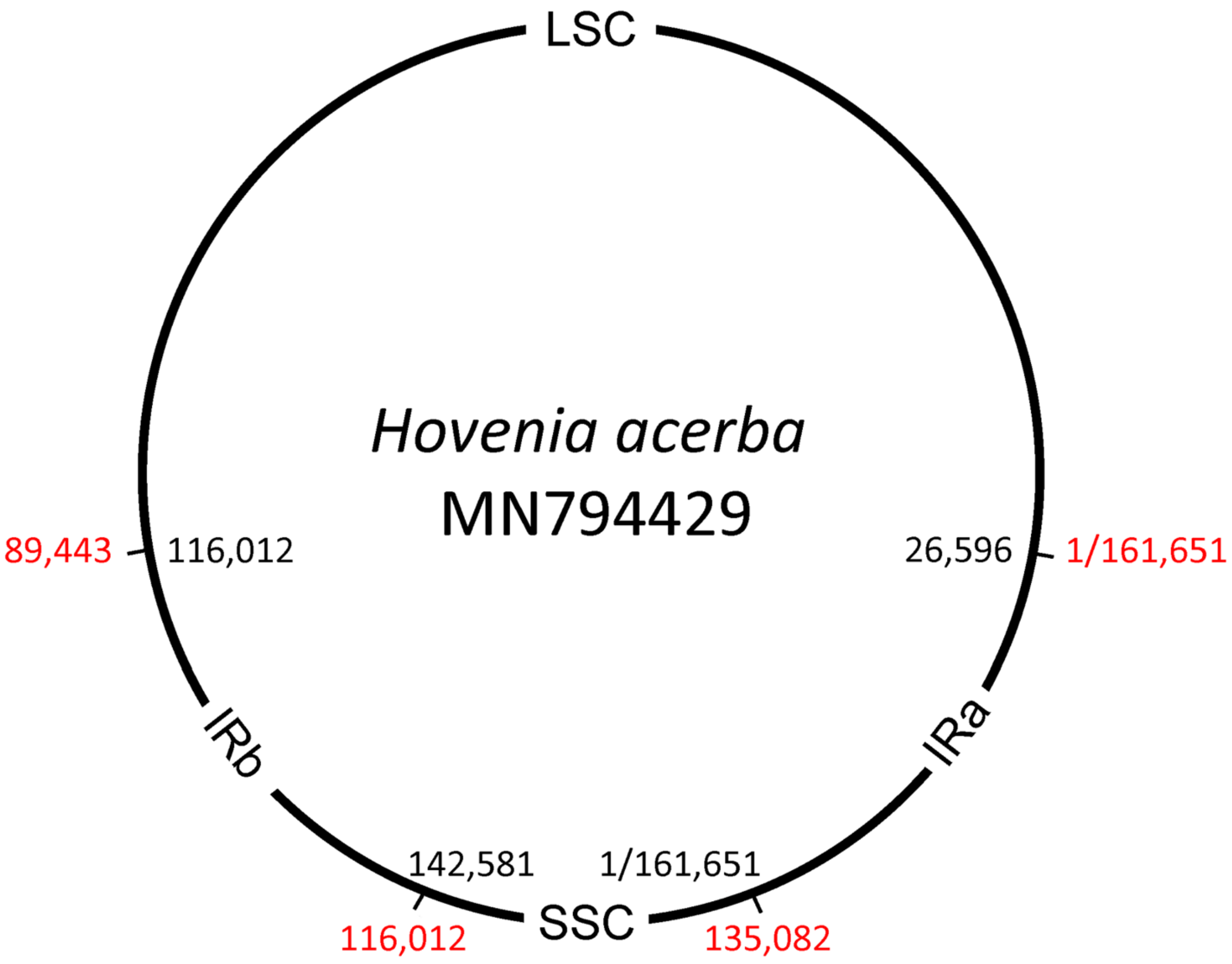

Figure S4. A shift in the origin of *H. acerba* MN794429 chloroplast genome and correction. Suggested origin is presented in red color.

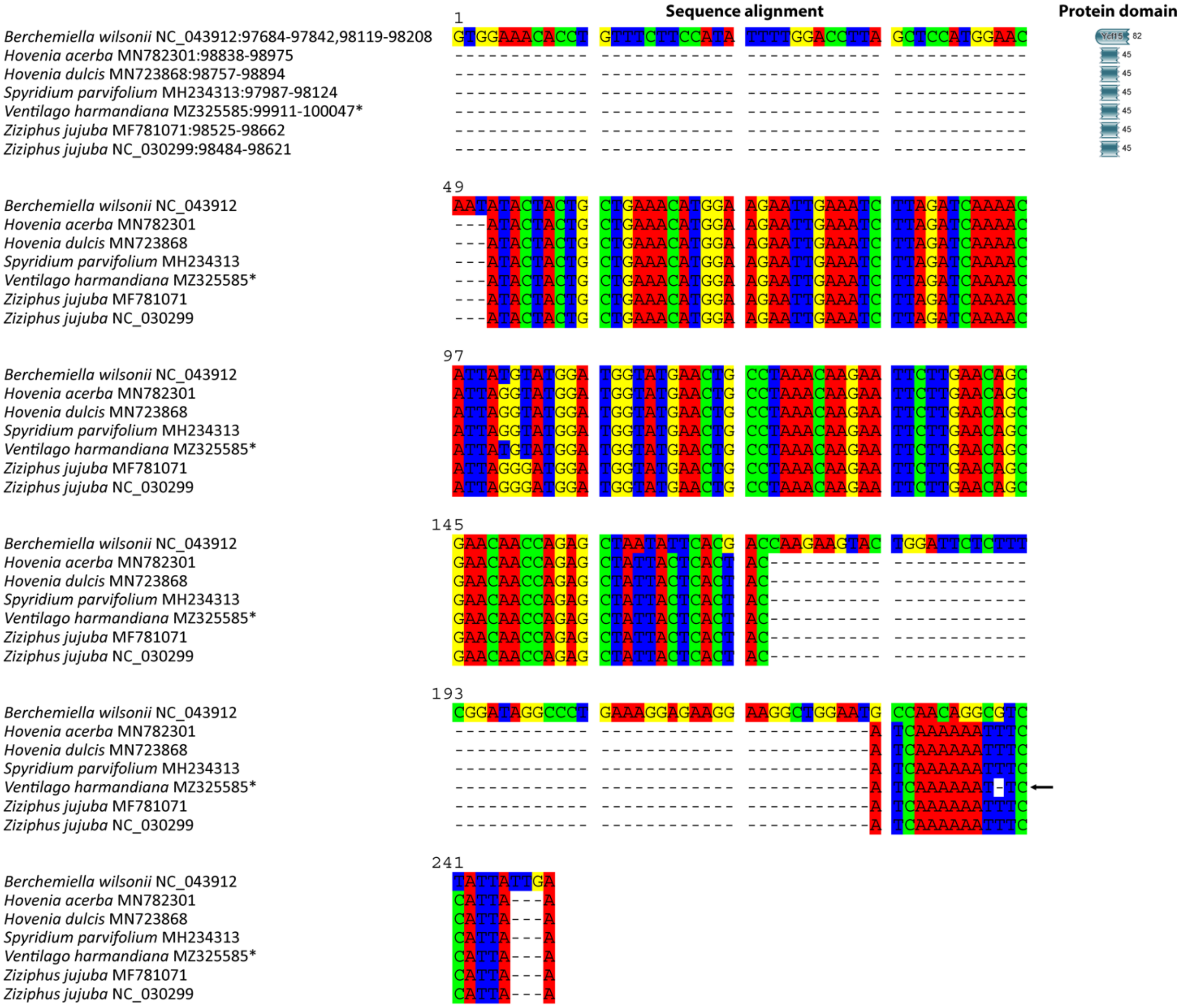

Figure S5. Multiple alignment of the *ycf15* gene sequences and matching domain fragments. This figure shows the alignment of the deposited *ycf15* DNA sequences from six Rhamnaceae organisms and the *ycf15* sequence of *V. harmandiana*. The predicted Ycf15 domain of each *ycf15* is illustrated on the right column. A missing T-base from the *ycf15* sequence of *V. harmandiana* is pointed out by an arrow. The full-length colored bar represents the presence of an entire Ycf15 domain. A truncated domain is shown by a jagged edge. The number indicates the amino acid length. Only the *ycf15* copy in IRb is shown, as its copy in IRa is identical.

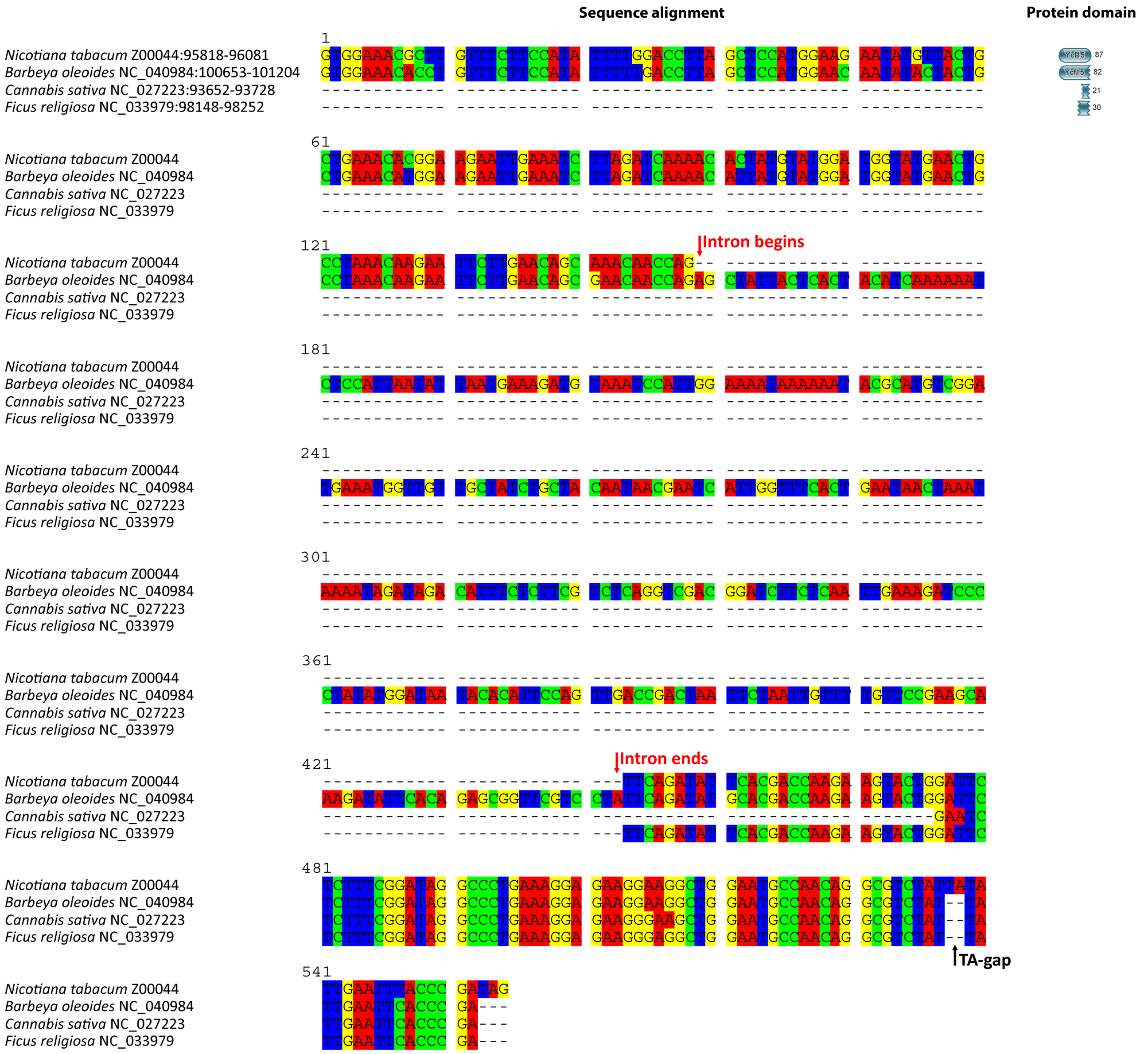

Figure S6. Re-annotation of the *ycf15* gene in *Barbeya oleoides* NC_040984 and the outgroup. The alignment of the *ycf15* gene sequences of *Barbeya oleoides*, *C. sativa* and *F. religiosa* to the *ycf15* gene sequence of *N. tabacum* Z00044 is illustrated. The TA-gap is pointed out by a black arrow. The predicted Ycf15 domain of each *ycf15* is illustrated on the right column. The full-length colored bar represents the presence of an entire Ycf15 domain. A truncated domain is shown by a jagged edge. The number indicates the amino acid length. Only the *ycf15* copy in IRb is shown, as its copy in IRa is identical.

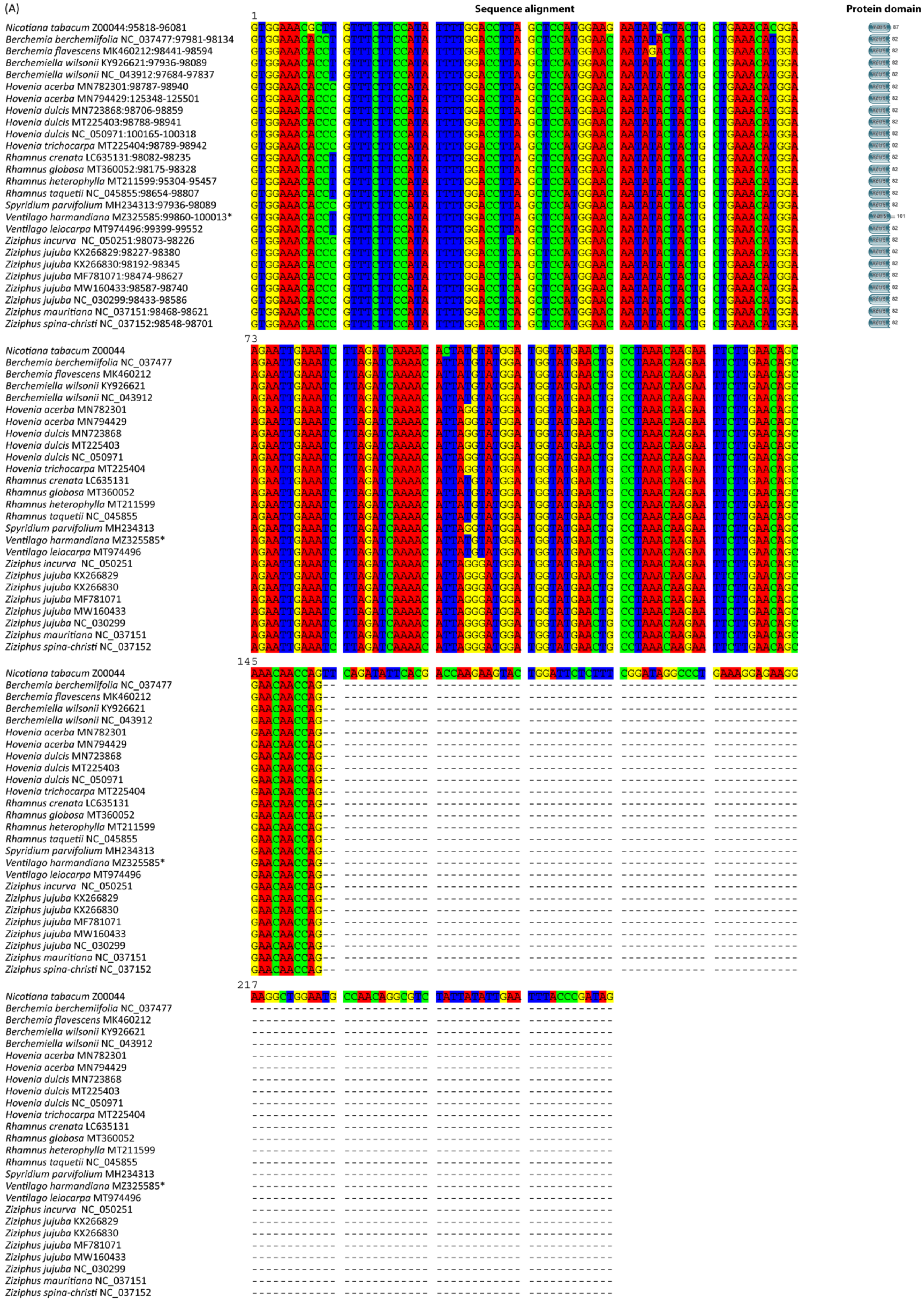
(A)
Sequence alignment
Protein domain
Nicotiana tabacum Z00044:95818-96081
Berchemia berchemiifolia NC_037477:97981-98134
Berchemia flavescens MK460212:98441-98594
Berchemiella wilsonii KY926621:97936-98089
Berchemiella wilsonii NC_043912:97684-97837
Hovenia acerba MN782301:98787-98940
Hovenia acerba MN794429:125348-125501
Hovenia dulcis MN723868:98706-98859
Hovenia dulcis MT225403:98788-98941
Hovenia dulcis NC_050971:100165-100318
Hovenia trichocarpa MT225404:98789-98942
Rhamnus crenata LC635131:98082-98235
Rhamnus globosa MT360052:98175-98328
Rhamnus heterophylla MT211599:95304-95457
Rhamnus taquetii NC_045855:98654-98807
Spyridium parvifolium MH234313:97936-98089
Ventilago harmandiana MZ325585:99860-100013*
Ventilago leiocarpa MT974496:99399-99552
Ziziphus incurva NC_050251:98073-98226
Ziziphus jujuba KX266829:98227-98380
Ziziphus jujuba KX266830:98192-98345
Ziziphus jujuba MF781071:98474-98627
Ziziphus jujuba MW160433:98587-98740
Ziziphus jujuba NC_030299:98433-98586
Ziziphus mauritiana NC_037151:98468-98621
Ziziphus spina-christi NC_037152:98548-98701

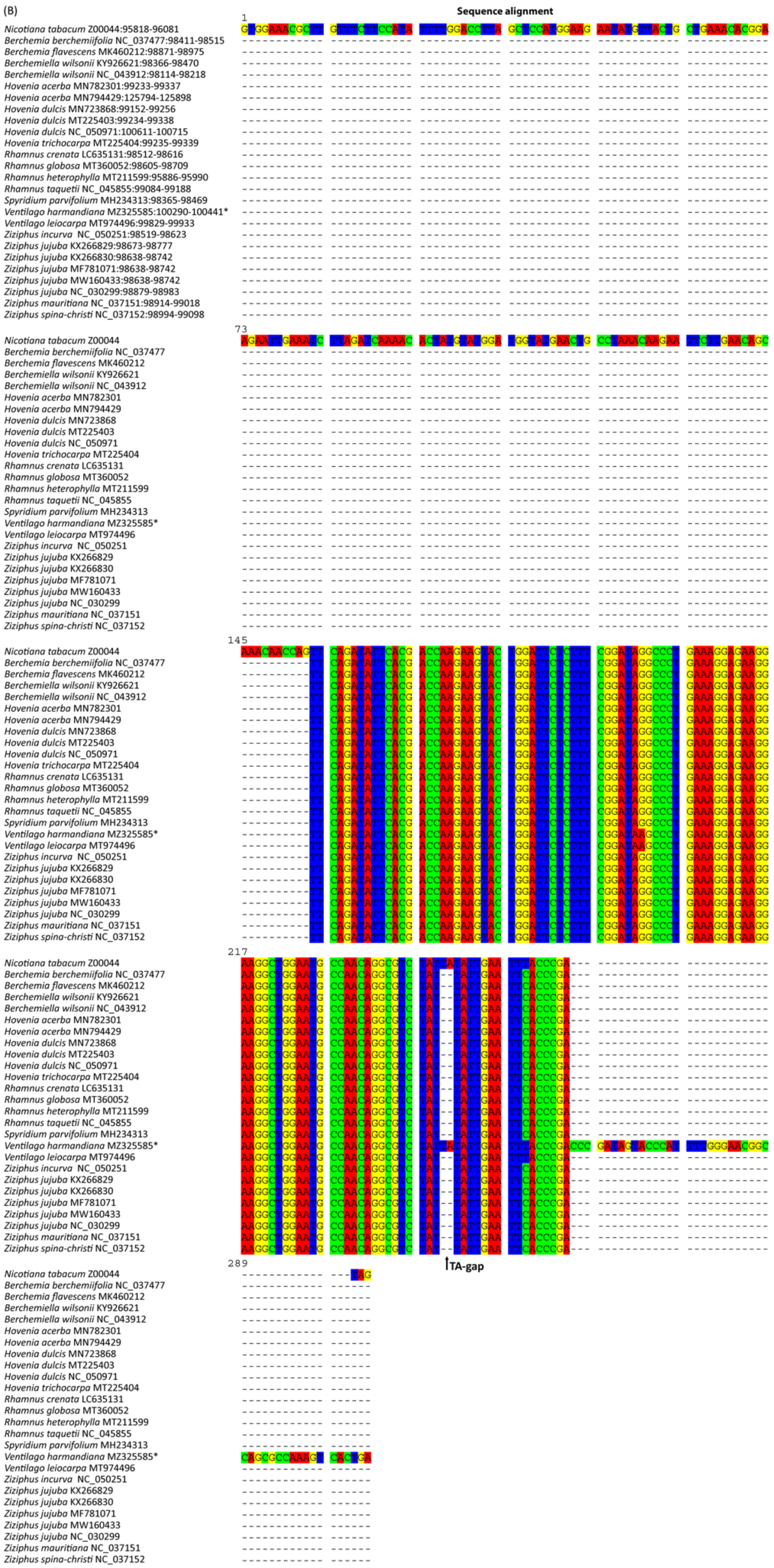
(B)
Sequence alignment
Nicotiana tabacum Z00044:95818-96081
Berchemia berchemiifolia NC_037477:98411-98515
Berchemia flavescens MK460212:98871-98975
Berchemiella wilsonii KY926621:98366-98470
Berchemiella wilsonii NC_043912:98114-98218
Hovenia acerba MN782301:99233-99337
Hovenia acerba MN794429:125794-125898
Hovenia dulcis MN723868:99152-99256
Hovenia dulcis MT225403:99234-99338
Hovenia dulcis NC_050971:100611-100715
Hovenia trichocarpa MT225404:99235-99339
Rhamnus crenata LC635131:98512-98616
Rhamnus globosa MT360052:98605-98709
Rhamnus heterophylla MT211599:95886-95990
Rhamnus taquetii NC_045855:99084-99188
Spyridium parvifolium MH234313:98365-98469
Ventilago harmandiana MZ325585:100290-100441*
Ventilago leiocarpa MT974496:99829-99933
Ziziphus incurva NC_050251:98519-98623
Ziziphus jujuba KX266829:98673-98777
Ziziphus jujuba KX266830:98638-98742
Ziziphus jujuba MF781071:98638-98742
Ziziphus jujuba MW160433:98638-98742
Ziziphus jujuba NC_030299:98879-98983
Ziziphus mauritiana NC_037151:98914-99018
Ziziphus spina-christi NC_037152:98994-99098
1
73
145
217
289
TA-gap

Figure S7. Re-annotation of the *ycf15* gene in Rhamnaceae. (A) The alignment of the reference gene sequence to the first exon and (B) to the second exon of the *ycf15* gene in Rhamnaceae organisms. The figure shows the multiple sequence alignment using *N. tabacum* Z00044 as a reference to re-annotate the *ycf15* gene of the organisms in this study. TA-gaps in the *ycf15* sequences of the Rhamnaceae are pointed out by a black arrow. The predicted Ycf15 domain of each *ycf15* is illustrated on the right column. The full-length colored bar represents the presence of an entire Ycf15 domain. A truncated domain is shown by a jagged edge. The number indicates the amino acid length. Only the *ycf15* copy in IRb is shown, as its copy in IRa is identical.

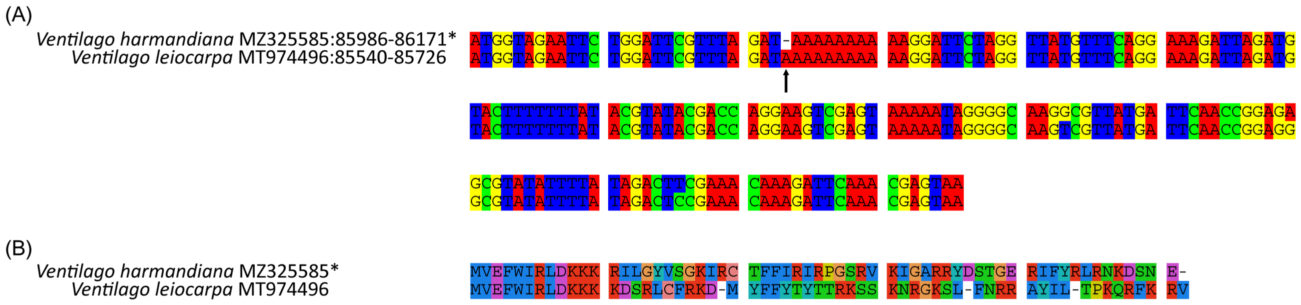

Figure S8. Re-annotation of *infA* gene in *V. leiocarpa*. (A) The alignment of *infA* DNA sequences between *V. harmandiana* and *V. leiocarpa*. A-gap is pointed out by an arrow. (B) Comparing amino acid sequences of the *infA* between *V. harmandiana* and *V. leiocarpa*. A dashed line (-) indicates a stop codon.

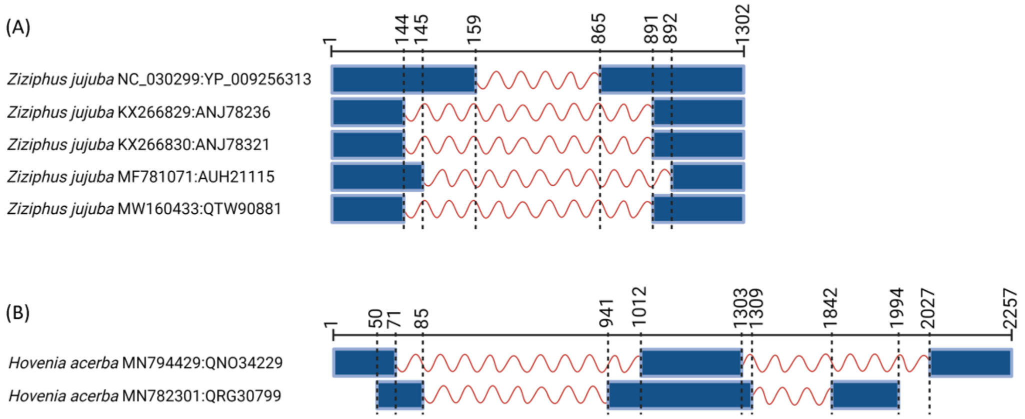

Figure S9. Comparisons of the *atpF* and *clpP* gene structures. (A) Comparing the *atpF* gene structure of *Z. jujuba* species. (B) Comparing the *clpP* gene structure of *H. acerba* species. A blue box represents a coding region and the red wavy line represents an intergenic region. The base position of the *atpF* in *Z. jujuba* NC_030299 and the *clpP* in *H. acerba* MN794429 is used as a reference for each gene.

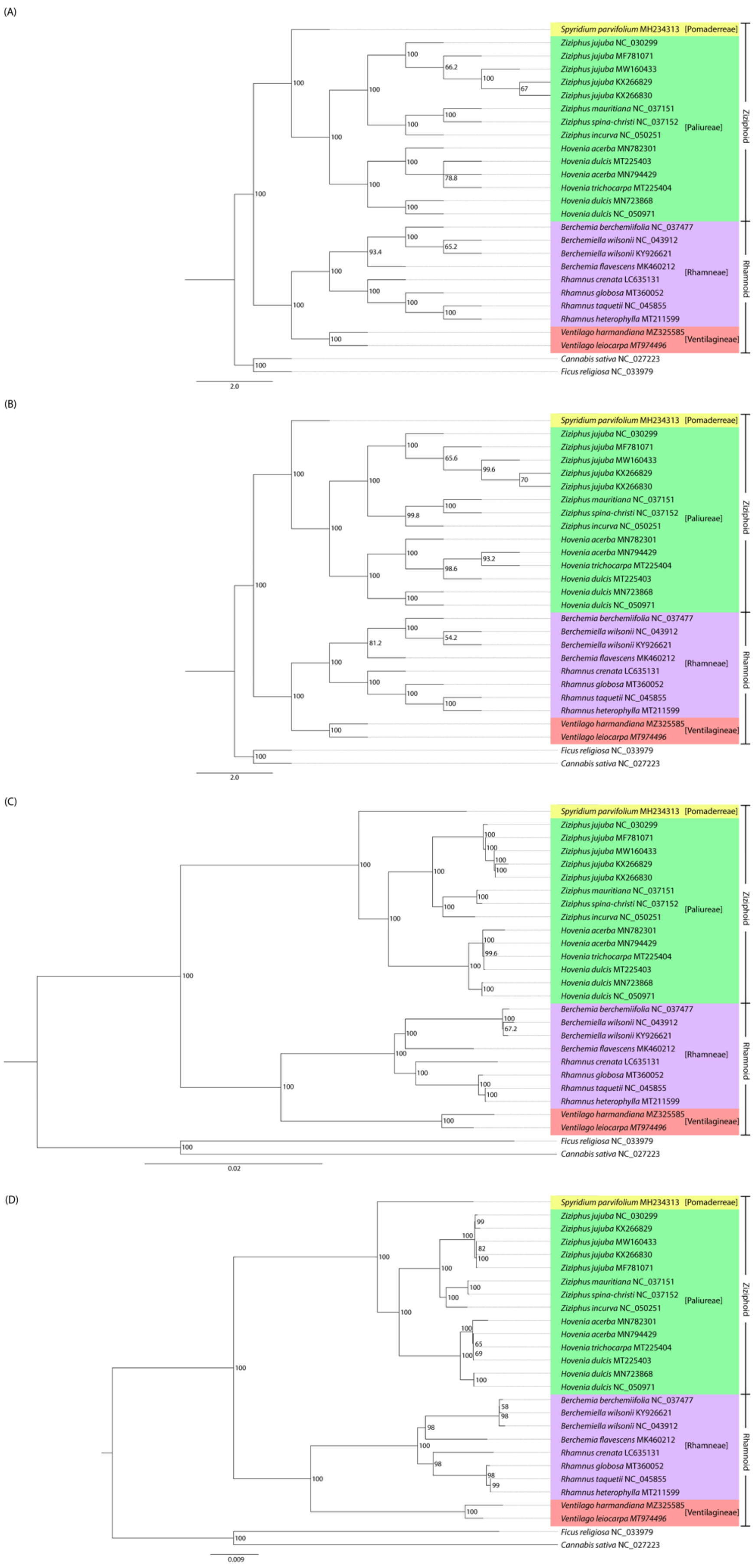
(A)
Spyridium parvifolium MH234313 [Pomaderreae]
Ziziphus jujuba NC_030299
Ziziphus jujuba MF781071
Ziziphus jujuba MW160433
Ziziphus jujuba KX266829
Ziziphus jujuba KX266830
Ziziphus mauritiana NC_037151
Ziziphus spina-christi NC_037152
Ziziphus incurva NC_050251
Hovenia acerba MN782301
Hovenia dulcis MT225403
Hovenia acerba MN794429
Hovenia trichocarpa MT225404
Hovenia dulcis MN723868
Hovenia dulcis NC_050971
[Paliureae]
Ziziphoid
Berchemia berchemiifolia NC_037477
Berchemiella wilsonii NC_043912
Berchemiella wilsonii KY926621
Berchemia flavescens MK460212
Rhamnus crenata LC635131
Rhamnus globosa MT360052
Rhamnus taquetii NC_045855
Rhamnus heterophylla MT211599
[Rhamneae]
Rhamnoid
Ventilago harmandiana MZ325585
Ventilago leiocarpa MT974496
[Ventilagineae]
Cannabis sativa NC_027223
Ficus religiosa NC_033979
2.0
(B)
Spyridium parvifolium MH234313 [Pomaderreae]
Ziziphus jujuba NC_030299
Ziziphus jujuba MF781071
Ziziphus jujuba MW160433
Ziziphus jujuba KX266829
Ziziphus jujuba KX266830
Ziziphus mauritiana NC_037151
Ziziphus spina-christi NC_037152
Ziziphus incurva NC_050251
Hovenia acerba MN782301
Hovenia acerba MN794429
Hovenia trichocarpa MT225404
Hovenia dulcis MT225403
Hovenia dulcis MN723868
Hovenia dulcis NC_050971
[Paliureae]
Ziziphoid
Berchemia berchemiifolia NC_037477
Berchemiella wilsonii NC_043912
Berchemiella wilsonii KY926621
Berchemia flavescens MK460212
Rhamnus crenata LC635131
Rhamnus globosa MT360052
Rhamnus taquetii NC_045855
Rhamnus heterophylla MT211599
[Rhamneae]
Rhamnoid
Ventilago harmandiana MZ325585
Ventilago leiocarpa MT974496
[Ventilagineae]
Ficus religiosa NC_033979
Cannabis sativa NC_027223
2.0
(C)
Spyridium parvifolium MH234313 [Pomaderreae]
Ziziphus jujuba NC_030299
Ziziphus jujuba MF781071
Ziziphus jujuba MW160433
Ziziphus jujuba KX266829
Ziziphus jujuba KX266830
Ziziphus mauritiana NC_037151
Ziziphus spina-christi NC_037152
Ziziphus incurva NC_050251
Hovenia acerba MN782301
Hovenia acerba MN794429
Hovenia trichocarpa MT225404
Hovenia dulcis MT225403
Hovenia dulcis MN723868
Hovenia dulcis NC_050971
[Paliureae]
Ziziphoid
Berchemia berchemiifolia NC_037477
Berchemiella wilsonii NC_043912
Berchemiella wilsonii KY926621
Berchemia flavescens MK460212
Rhamnus crenata LC635131
Rhamnus globosa MT360052
Rhamnus taquetii NC_045855
Rhamnus heterophylla MT211599
[Rhamneae]
Rhamnoid
Ventilago harmandiana MZ325585
Ventilago leiocarpa MT974496
[Ventilagineae]
Ficus religiosa NC_033979
Cannabis sativa NC_027223
0.02
(D)
Spyridium parvifolium MH234313 [Pomaderreae]
Ziziphus jujuba NC_030299
Ziziphus jujuba KX266829
Ziziphus jujuba MW160433
Ziziphus jujuba KX266830
Ziziphus jujuba MF781071
Ziziphus mauritiana NC_037151
Ziziphus spina-christi NC_037152
Ziziphus incurva NC_050251
Hovenia acerba MN782301
Hovenia acerba MN794429
Hovenia trichocarpa MT225404
Hovenia dulcis MT225403
Hovenia dulcis MN723868
Hovenia dulcis NC_050971
[Paliureae]
Ziziphoid
Berchemia berchemiifolia NC_037477
Berchemiella wilsonii KY926621
Berchemiella wilsonii NC_043912
Berchemia flavescens MK460212
Rhamnus crenata LC635131
Rhamnus globosa MT360052
Rhamnus taquetii NC_045855
Rhamnus heterophylla MT211599
[Rhamneae]
Rhamnoid
Ventilago harmandiana MZ325585
Ventilago leiocarpa MT974496
[Ventilagineae]
Ficus religiosa NC_033979
Cannabis sativa NC_027223
0.009

60

Figure S10. Phylogenetic trees of Rhamnaceae reconstructed from the orthologous proteins (A) using ML method, (B) using MP method, (C) using BI method and (D) the tree reconstructed from the orthologous genes using RAxML method. Different colors correspond to tribes labelled in square brackets. The outgroup branch is not colored. Two major groups of Rhamnaceae: ziziphoid and rhamnoid are presented on the rightmost text. A number in each tree indicates the ML bootstrap value, the MP bootstrap value, the posterior probability of BI, the ML bootstrap value, respectively.

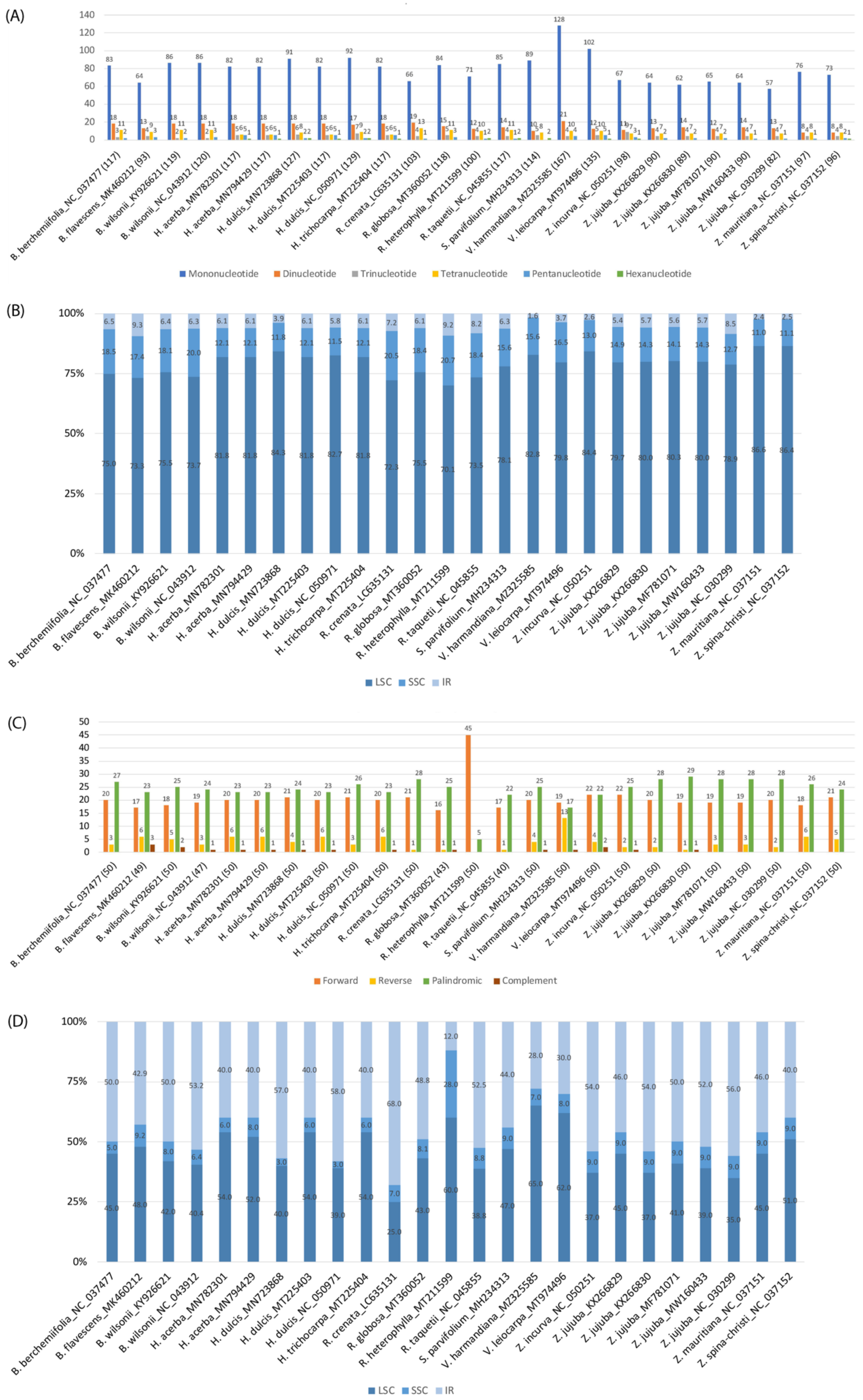
(A)
B. berchemiifolia_NC_037477 (117)
B. flavescens_MK460212 (93)
B. wilsonii_KY926621 (119)
B. wilsonii_NC_043912 (120)
H. acerba_MN782301 (117)
H. acerba_MN794429 (117)
H. dulcis_MN723868 (127)
H. dulcis_MT225403 (117)
H. dulcis_NC_050971 (129)
H. trichocarpa_MT225404 (117)
R. crenata_LC635131 (103)
R. globosa_MT360052 (118)
R. heterophylla_MT211599 (100)
R. taquetii_NC_045855 (117)
S. parvifolium_MH234313 (114)
V. harmandiana_MZ325585 (167)
V. leiocarpa_MT974496 (135)
Z. incurva_NC_050251 (98)
Z. jujuba_KX266829 (90)
Z. jujuba_KX266830 (89)
Z. jujuba_MF781071 (90)
Z. jujuba_MW160433 (90)
Z. jujuba_NC_030299 (82)
Z. mauritiana_NC_037151 (97)
Z. spina-christi_NC_037152 (96)
Mononucleotide
Dinucleotide
Trinucleotide
Tetranucleotide
Pentanucleotide
Hexanucleotide
(B)
LSC
SSC
IR
(C)
B. berchemiifolia_NC_037477 (50)
B. flavescens_MK460212 (49)
B. wilsonii_KY926621 (50)
B. wilsonii_NC_043912 (47)
H. acerba_MN782301 (50)
H. acerba_MN794429 (50)
H. dulcis_MN723868 (50)
H. dulcis_MT225403 (50)
H. dulcis_NC_050971 (50)
H. trichocarpa_MT225404 (50)
R. crenata_LC635131 (50)
R. globosa_MT360052 (43)
R. heterophylla_MT211599 (50)
R. taquetii_NC_045855 (40)
S. parvifolium_MH234313 (50)
V. harmandiana_MZ325585 (50)
V. leiocarpa_MT974496 (50)
Z. incurva_NC_050251 (50)
Z. jujuba_KX266829 (50)
Z. jujuba_KX266830 (50)
Z. jujuba_MF781071 (50)
Z. jujuba_MW160433 (50)
Z. jujuba_NC_030299 (50)
Z. mauritiana_NC_037151 (50)
Z. spina-christi_NC_037152 (50)
Forward
Reverse
Palindromic
Complement
(D)
LSC
SSC
IR

Figure S11. Characteristics and comparisons of Rhamnaceae chloroplast genome features. (A) Comparison of the SSR types. (B) Distribution of SSRs in LSC, SSC and IR regions. (C) Comparison of the long repeat types. (D) Distribution of long repeats in LSC, SSC and IR regions. The total number of SSRs and long repeat sequences in each species is in the parenthesis.

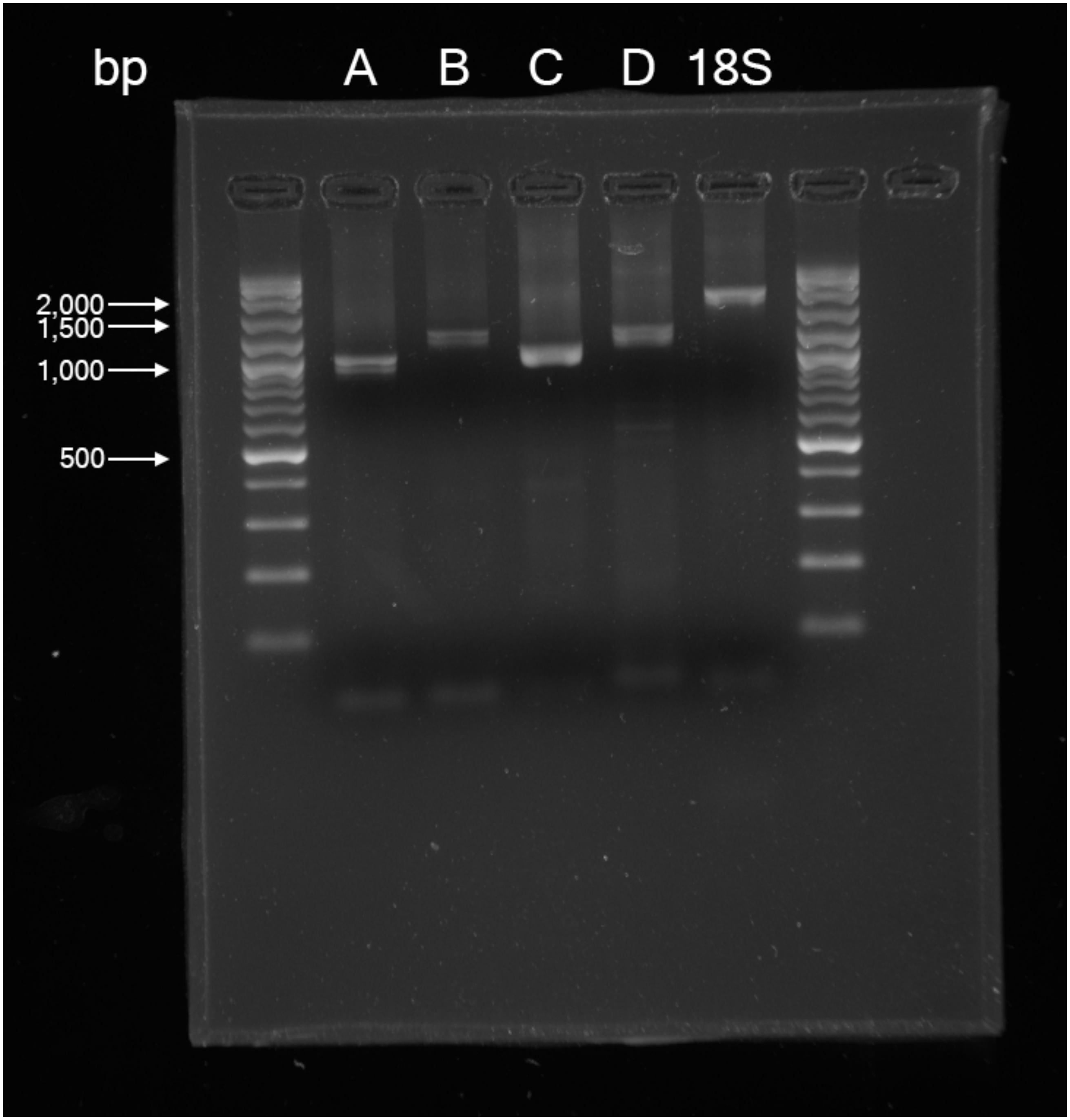

Figure S12. The original image of gel in Figure 1C before cropping and inverting color.
