# Supplementary material for: Revisiting chloroplast genomic landscape and annotation towards comparative chloroplast genomes of Rhamnaceae
Source: BMC Plant Biol. 2023 Jan 28;23:59. doi: 10.1186/s12870-023-04074-5 (PMC9883906; doi:10.1186/s12870-023-04074-5)
Supplement: Supplementary file 2 — Additional file 2: Table S1. List of primers. Table S2. List of 25 Rhamnaceae complete chloroplast genomes and the outgroup for the phylogenetic analysis. Table S3. List of re-annotated genes. Table S4. List of orthologous genes for phylogenetic reconstruction. [file 12870_2023_4074_MOESM2_ESM.pdf]

Table S1. List of primers.

Primer sets	Primers	Primer Sequence (5’-3’)	Length	GC (%)	T_m* (°C)	Expected size (bp)
VharA	VharA-F	GTG CCC TCG TTG ACT TCA GC	20	60.0	58.7	951
	VharA-R	GCA ACA GTC GGA CAA GTG GG	20	60.0	58.8	
VharB	VharB-F	TTA GGC AGA ATA CCG TCA CCC	20	52.4	56.6	1,189
	VharB-R	GTA AGA AGC ATG ACG CAG CC	20	55.0	56.5	
VharC	VharC-F	TAA CAA GCT GGG TGG GTA TGT	21	47.6	56.1	1,035
	VharC-R	GTG CCT CTG CAT CTA GCA TTG	20	52.4	56.2	
VharD	VharD-F	GCA ACA GTC GGA CAA GTG GG	20	60.0	58.8	1,193
	VharD-R	CCC TCT AGA CCT AGC TGC TGT C	22	59.1	58.4	
VharIR1	VharIR1-F	GTC TTC CCG TTC CTT CAT CTG	21	52.4	55.7	1,039
	VharIR1-R	GGG TCG AAC TCT TCT TTG GTG	21	52.4	55.7	
VharIR2	VharIR2-F	CAG GGT TCC ACA AGC AAT CG	20	55.0	56.6	918
	VharIR2-R	ACG ATA CAT TCC GTA CCT TGC	21	47.6	54.8	
VharIR3	VharIR3-F	ACT CTA TCA ATA ACC GAG CCG	21	47.6	54.1	857
	VharIR3-R	GCC CTA TGA ATG GGG ATA TTC C	22	50.0	55.1	
VharIR4	VharIR4-F	TCC TGT TTA GCA GAA AGA CGG	21	47.6	54.7	1,056

Primer sets	Primers	Primer Sequence (5'-3')	Length	GC (%)	T_m* (^oC)	Expected size (bp)
	VharIR4-R	TCA TGC TCG TTC CAA GTT CG	20	50.0	55.4	
VharIR5	VharIR5-F	TTG GGC TCC TAG AAT ATG GC	20	50.0	54.2	949
	VharIR5-R	AAC CAT TTC ATC CGA CAG GC	20	50.0	55.6	
VharIR6	VharIR6-F	CGG GTT CAT TGA TAT TCC TGG	21	47.6	53.3	972
	VharIR6-R	CTG ACT TGC TCC TAC GGA AC	20	55.0	55.2	
VharIR7	VharIR7-F	ACT CCA CGT ATT GCT TGA CG	20	50.0	55.1	851
	VharIR7-R	AGG AAC CCT AGA TGC TGT CG	20	55.0	56.3	
VharIR8	VharIR8-F	TCA ATG AGC GAA AGG TAC GAA	21	42.9	54.2	982
	VharIR8-R	GTT CTT ATG TGT TTC CAC GCC	21	47.6	54.3	
VharIR9	VharIR9-F	TGG TGG GGT TAC CAT TAT CC	20	50.0	54.2	938
	VharIR9-R	TCC GAC TTG CAT GTG TTA AGC	21	47.6	55.7	
VharIR10	VharIR10-F	TTG AAA GAG AGG GGT GCC TT	20	50.0	56.3	967
	VharIR10-R	AGT ACG CCA TGC TAA TGT GC	20	50.0	55.6	
VharIR11	VharIR11-F	GAC AGG CTA TTA GCT CAG TGG	21	52.4	54.9	891
	VharIR11-R	GAT TCA TCC TTC CCG AAC GC	20	55.0	56.1	
VharIR12	VharIR12-F	TGC CTG TTG AAG AAT GAG CC	20	50.0	55.5	909

Primer sets	Primers	Primer Sequence (5'-3')	Length	GC (%)	T_m* (°C)	Expected size (bp)
	VharIR12-R	CAC CTT GCG TCC TTG AAC C	19	57.9	56.5	
VharIR13	VharIR13-F	GAG AGG CTC GGT GAA ATA GAC	21	52.4	54.8	962
	VharIR13-R	CAC TTC CAC TTG ACA CCT ATC G	22	50.0	55.2	
VharIR14	VharIR14-F	GGG CAG AAT AGC GGA GAA AG	20	55.0	55.6	1,014
	VharIR15-R	GGT CTA CCC AAT GCT AGA TGC	21	52.4	55.2	

* Melting temperatures were calculated using OligoAnalyzer™ (Integrated DNA Technologies, USA) with 0.25 µM Oligo concentration and 50 mM Na^+ concentration.

VharA–D primers are for validation of the IR boundaries and VharIR1–14 primers are for correction of mismatched nucleotide bases between IRs

Table S2. List of 25 Rhamnaceae complete chloroplast genomes and the outgroup for the phylogenetic analysis.

Species	GenBank accession number	Size (bp)	Number of genes	Protein coding gene	tRNA	rRNA	GC (%)	LSC (bp)	IR (bp)	SSC (bp)	Reference
Berchemia berchemiifolia (Berchemiella berchemiifolia)	NC_037477	160410	127 (133)	82 (88)	37	8	37.2	88627	26514	18755	(1)
Berchemia flavescens	MK460212	160872	129 (133)	84 (88)	37	8	37.2	89066	26526	18754	(2)
Berchemiella wilsonii	NC_043912	160076	127 (133)	83 (88)	36 (37)	8	37.2	88331	26514	18717	(3)
Berchemiella wilsonii var. wilsonii	KY926621	160333	129 (133)	84 (88)	37	8	37.2	88582	26510	18731	(4)
Hovenia acerba	MN794429	161651	121 (133)	78 (88)	35 (37)	8	36.7	89443	26569	19070	(5)
Hovenia acerba	MN782301	161668	130 (133)	85 (88)	37	8	36.7	89452	26618	18980	(6)
Hovenia dulcis	NC_050971	162962	130 (133)	85 (88)	37	8	36.6	90900	26571	18920	(7)
Hovenia dulcis	MT225403	161667	121 (133)	78 (88)	35 (37)	8	36.7	89455	26616	18980	(8)
Hovenia dulcis voucher lpssy0298	MN723868	161636	131 (133)	86 (88)	37	8	36.6	89839	26316	19165	(7)
Hovenia trichocarpa	MT225404	161668	121 (133)	78 (88)	35 (37)	8	36.7	89455	26617	18979	(8)

Species	GenBank accession number	Size (bp)	Number of genes	Protein coding gene	tRNA	rRNA	GC (%)	LSC (bp)	IR (bp)	SSC (bp)	Reference
Rhamnus crenata Rhcr	LC635131	160454	126 (132)	84 (87)	34 (37)	8	37.0	88885	26418	18733	(9)
Rhamnus globosa voucher HCY2019012	MT360052	160642	129 (133)	84 (88)	37	8	37.1	88889	26428	18897	(10)
Rhamnus heterophylla	MT211599	156514	129 (133)	84 (88)	37	8	37.3	86024	25305	19880	(11)
Rhamnus taquetii	NC_045855	161205	129 (133)	84 (88)	37	8	37.1	89373	26448	18936	(12)
Spyridium parvifolium var. parvifolium isolate CC211	MH234313	161012	130 (133)	86 (88)	36 (37)	8	36.9	88814	26515	19168	(13)
***Ventilago harmandiana* Pierre**	MZ325585	162904	134	89	37	8	36.6	90807	26401	19295	**This study**
Ventilago leiocarpa	MT974496	161880	127 (134)	82 (89)	37	8	37.0	90241	26255	19129	(14)
Ziziphus incurva	NC_050251	160920	129 (133)	84 (88)	37	8	36.8	88778	26477	19188	(15)
Ziziphus jujuba	NC_030299	161466	129 (134)	85 (89)	36 (37)	8	36.8	89120	26499	19348	(16)
Ziziphus jujuba cultivar Dongzao voucher BZG2016009	MF781071	161493	130 (134)	85 (89)	37	8	36.8	89178	26479	19357	(17)
Ziziphus jujuba var. spinosa	MW160433	161606	130 (134)	85 (89)	37	8	36.8	89292	26479	19356	(18)

Species	GenBank accession number	Size (bp)	Number of genes	Protein coding gene	tRNA	rRNA	GC (%)	LSC (bp)	IR (bp)	SSC (bp)	Reference
Ziziphus jujuba var. spinosa voucher Suanzao-007	KX266830	161211	130 (134)	85 (89)	37	8	36.8	88898	26478	19357	(19)
Ziziphus jujuba voucher Junzao-001	KX266829	161215	130 (134)	85 (89)	37	8	36.8	88975	26435	19370	(19)
Ziziphus mauritiana voucher ZMAU20161128	NC_037151	161543	129 (134)	84 (89)	37	8	36.8	89081	26558	19346	(19)
Ziziphus spina-christi voucher ZSC20161128	KY628305	161615	129 (134)	84 (89)	37	8	36.8	89161	26558	19338	(19)
Cannabis sativa cultivar Yoruba Nigeria*	NC_027223	153854	131 (133)	86 (88)	37	8	36.7	84028	26009	17808	(20)
Ficus religiosa voucher Ronsted86*	NC_033979	160627	130 (133)	85 (88)	37	8	35.9	88815	25853	20106	(21)

* Outgroups in the phylogeny
Numbers in parentheses indicate the number of genes after re-annotation.

References

1. Cheon KS, Kim KA, Yoo KO. The complete chloroplast genome sequence of Berchemia berchemiifolia (Rhamnaceae). Mitochondrial DNA B Resour. 2018;3(1):133-4.
2. Zhu X-F, Li Y, Lu Z. The complete chloroplast genome sequence of Berchemia flavescens (Rhamnaceae). Mitochondrial DNA Part B. 2019;4(1):1302-3.
3. Li Y, Wang J, Li P, Cheng S, Wang F. The complete chloroplast genome sequence of Berchemiella wilsonii (Rhamnaceae), an endangered endemic species. Mitochondrial DNA Part B. 2019;4(1):452-4.
4. Wang Y-H, Chen S-Y, Zhang S-D. Characterization of the complete chloroplast genome of Berchemiella wilsonii var. wilsonii (Rhamnaceae), an endangered species endemic to China. Conservation Genetics Resources. 2018;10(1):39-41.
5. Zhang L, Mao R, Bi H, Shen J, Wang Y, Li M. Characterization of the complete chloroplast genome of Hovenia acerba (Rhamnaceae). Mitochondrial DNA B Resour. 2020;5(1):934-5.

6. Yin Y, Tao J, Yang X, Jiao Q, Li Y, Feng L. The first complete chloroplast genome of Hovenia acerba Lindl. Mitochondrial DNA B Resour. 2020;5(1):658-9.
7. Ling L-Z, Zhang S-D. The complete chloroplast genome of Hovenia dulcis (Rhamnaceae). Mitochondrial DNA Part B. 2020;5(1):665-6.
8. Li M, Ye X, Bi H. Characterization of the complete chloroplast genome of two Hovenia species (Rhamnaceae). Mitochondrial DNA Part B. 2020;5(2):1731-2.
9. Wang J, Yang S. The complete chloroplast genome of Rhamnus crenata Siebold & Zuccarini (Rhamnaceae). Mitochondrial DNA Part B, Resources. 2021;6(9):2489-90.
10. Xie Y, Wang Z, Jiang X, Zhang X. The complete chloroplast genome of Rhamnus globosa (Rhamnaceae). Mitochondrial DNA B Resour. 2020;5(3):2830-1.
11. Li B, Chen H, Chen J. The complete chloroplast genome of plant Rrhamnus heterophylla (Rhamnaceae). Mitochondrial DNA Part B. 2020;5(2):1850-1.
12. Jin D-P, Park J-W, Park J-S, Choi B-H. The complete plastid genome of Rhamnus taquetii, an endemic shrub on the Jeju Island of Korea. Mitochondrial DNA Part B. 2020;5(1):924-6.
13. Clowes C, Fowler RM, Brown GK, Bayly MJ. The complete chloroplast genome sequence of Spyridium parvifolium var. parvifolium (family Rhamnaceae; tribe Pomaderreae). Mitochondrial DNA Part B. 2018;3(2):807-9.
14. Lu X, Luo Q, Qin Y, Yan Q, Guo S. The complete chloroplast genome sequence of Ventilago leiocarpa Benth. Mitochondrial DNA Part B. 2021;6(3):736-7.
15. Wang Y, Hao J, Yuan X, Lu B. The complete chloroplast genome sequence of Ziziphus incurva. Mitochondrial DNA Part B, Resources. 2019;4(2):3465-6.
16. Ma Q, Li S, Bi C, Hao Z, Sun C, Ye N. Complete chloroplast genome sequence of a major economic species, Ziziphus jujuba (Rhamnaceae). Curr Genet. 2017;63(1):117-29.
17. Gao C-M, Gao Y-C, Liu X-H. The complete genome of Ziziphus jujuba cv. dongzao, an economic crop in Yellow River Delta of China. Mitochondrial DNA Part B. 2017;2(2):692-3.
18. Zhang Y, Hu G, Mao W, Dong N, Chen B, Pan Q. Chloroplast genome sequence of the wild Ziziphus jujuba Mill. var. spinosa from North China. Mitochondrial DNA B Resour. 2021;6(2):666-7.
19. Huang J, Chen R, Li X. Comparative Analysis of the Complete Chloroplast Genome of Four Known Ziziphus Species. Genes (Basel). 2017;8(12).
20. Oh H, Seo B, Lee S, Ahn DH, Jo E, Park JK, et al. Two complete chloroplast genome sequences of Cannabis sativa varieties. Mitochondrial DNA A DNA Mapp Seq Anal. 2016;27(4):2835-7.
21. Bruun-Lund S, Clement WL, Kjellberg F, Rønsted N. First plastid phylogenomic study reveals potential cyto-nuclear discordance in the evolutionary history of Ficus L. (Moraceae). Molecular Phylogenetics and Evolution. 2017;109:93-104.

Table S3. List of re-annotated genes.

Species	Gene	Updated position	Detail
Berchemia berchemiifolia NC_037477	*rps19*	complement(88822..88544)	Re-annotate a missing gene in IRb
	rps19	join(160216..160410,1..33)	Re-annotate a missing gene in IRa
	ycf1	complement(134970..129272)	Re-annotate a missing gene in IRa
	ycf1	114068..115147	Re-annotate a missing gene in IRb
	ycf15	join(97981..98134,98411..98515)	Re-annotate a missing gene in IRb
	ycf15	complement(join(150523..150627,150904..151057))	Re-annotate a missing gene in IRa
Berchemia flavescens MK460212	*ndhB*	complement(join(99945..100700,101381..102157))	Correct gene location
	ndhB	join(147781..148557,149238..149993)	Correct gene location
	ndhF	complement(115617..117857)	Correct gene location
	psbJ	complement(68137..68259)	Correct gene location
	psbK	7782..7967	Correct gene location
	rpoC1	complement(join(22420..24030,24811..25242))	Correct gene location
	rpoC2	complement(18085..22242)	Correct gene location
	rps19	join(160678..160872,1..24)	Re-annotate a missing gene in IRa
	ycf1	114510..115598	Re-annotate a missing gene in IRb
	ycf15	join(98441..98594,98871..98975)	Re-annotate a missing gene in IRb
	ycf15	complement(join(150964..151068,151345..151498))	Re-annotate a missing gene in IRa
	ycf4	64488..65042	Correct gene location

Species	Gene	Updated position	Detail
Berchemiella wilsonii KY926621	*rps19*	join(160139..160333,1..24)	Re-annotate a missing gene in IRa
	ycf1	114027..115097	Re-annotate a missing gene in IRb
	ycf15	join(97936..98089,98366..98470)	Re-annotate a missing gene in IRb
	ycf15	complement(join(150446..150550,150827..150980))	Re-annotate a missing gene in IRa
Berchemiella wilsonii NC_043912	*atpF*	complement(join(14287..14142,13353..12943))	Re-annotate a missing gene in LSC
	ndhD	complement(120550..122061)	Correct gene location
	ndhK	complement(53244..53924)	Correct gene location
	petB	join(79057..79062,79850..80491)	Correct gene location
	petD	join(80684..80690,81411..81886)	Re-annotate a missing gene in LSC
	petN	30714..30803	Correct gene location
	psbC	36619..38040	Correct gene location
	psbI	8491..8601	Correct gene location
	psbK	7856..8041	Correct gene location
	psbL	complement(67927..67811)	Re-annotate a missing gene in LSC
	psbN	complement(78152..78593)	Correct gene location
	rbcL	58808..60235	Correct gene location
	rpl36	complement(83821..83708)	Correct sequence
	rpoC1	complement(24770..25204)	Correct gene location
	rpoC2	complement(18037..22209)	Correct gene location

Species	Gene	Updated position	Detail
	rps16	complement(join(6674..6713,5794..5558))	Re-annotate a missing gene in LSC
	rps19	join(159882..160076,1..24)	Correct gene location
	trnG-UCC	join(9954..9975,10693..10739)	Re-annotate a missing gene in LSC
	ycf15	join(97684..97837,98114..98218)	Correct gene location
	ycf15	complement(join(150190..150294,150571..150724))	Correct gene location
Hovenia acerba MN782301	*ndhD*	complement(122015..123526)	Correct gene location
	psbL	complement(68666..68550)	Re-annotate a missing gene in LSC
	psbN	complement(79339..79470)	Correct gene location
	rpoC1	complement(join(22540..24165,24924..25353))	Correct gene location
	rps19	join(161535..161668,1..10)	Re-annotate a missing gene in IRa
	ycf1	114855..116090	Re-annotate a missing gene in IRb
	ycf15	join(98787..98940,99233..99337)	Correct gene location
	ycf15	complement(join(151783..151887,152180..152333))	Correct gene location
Hovenia acerba MN794429	*atpF*	complement(join(41094..40949,40213..39803)	Re-annotate a missing gene in LSC
	ndhD	complement(150078..148567)	Re-annotate a missing gene in SSC
	petD	join(108190..108196,108934..109409)	Re-annotate a missing gene in LSC
	psbC	63230..64650	Re-annotate a missing gene in LSC
	psbL	complement(95227..95111)	Re-annotate a missing gene in LSC

Species	Gene	Updated position	Detail
	rpoC2	complement(44768..48931)	Correct gene location
	rps19	complement(116146..115868)	Re-annotate a missing gene in IRb
	rps19	join(26436..26569,1..10)	Re-annotate a missing gene in IRa
	trnfM-CAU	66343..66416	Re-annotate a missing gene in LSC
	trnG-UCC	join(36504..36526,37230..37278)	Re-annotate a missing gene in LSC
	ycf1	141416..142651	Re-annotate a missing gene in IRb
	ycf1	complement(join(157061..161651,1..1166))	Correct gene location
	ycf15	join(16684..16788,17081..17234)	Re-annotate a missing gene in IRa
	ycf15	complement(join(125348..125501,125794..125898))	Re-annotate a missing gene in IRb
Hovenia dulcis MN723868	*ndhE*	complement(124050..124355)	Correct gene location
	psbJ	complement(68344..68466)	Correct gene location
	rpoA	complement(82931..83926)	Correct gene location
	rpoC1	complement(join(22437..24047,24811..25242))	Correct gene location
	rpoC2	complement(18105..22259)	Correct gene location
	rps19	161458..161505	Re-annotate a missing gene in IRa
	ycf1	114776..116023	Re-annotate a missing gene in IRb
	ycf15	join(98706..98859,99152..99256)	Correct gene location
	ycf15	complement(join(151689..151793,152086..152239))	Correct gene location

Species	Gene	Updated position	Detail
Hovenia dulcis MT225403	*atpF*	complement(join(14382..14527,13647..13237))	Re-annotate a missing gene in LSC
	ndhD	complement(123527..122016)	Re-annotate a missing gene in SSC
	petD	(81630..81636),(82374..82849)	Re-annotate a missing gene in LSC
	psbC	36669..38089	Re-annotate a missing gene in LSC
	psbL	complement(68668..68552)	Re-annotate a missing gene in LSC
	rpoC2	complement(18201..22364)	Correct gene location
	rps19	complement(89586..89308)	Re-annotate a missing gene in IRb
	rps19	join(161536..161667,1..12)	Re-annotate a missing gene in IRa
	trnfM-CAU	39782..39855	Re-annotate a missing gene in LSC
	trnG-UCC	join(9937..9959,10665..10712)	Re-annotate a missing gene in LSC
	ycf1	complement(join(130510..132533,132567..136266))	Correct gene location
	ycf1	114856..116091	Re-annotate a missing gene in IRb
	ycf15	join(98788..98941,99234..99338)	Re-annotate a missing gene in IRb
	ycf15	complement(join(151784..151888,152181..152334))	Re-annotate a missing gene in IRa
Hovenia dulcis NC_050971	*ndhB*	complement(join(101700..102455,103136..103912))	Correct gene location
	ndhB	join(149951..150727,151408..152163)	Correct gene location
	ndhD	complement(123381..124892)	Correct gene location
	ndhE	complement(125509..125814)	Correct gene location

Species	Gene	Updated position	Detail
	psbJ	complement(69803..69925)	Correct gene location
	psbZ	38961..39149	Correct gene location
	rpoA	complement(84390..85385)	Correct gene location
	rpoC1	complement(join(22631..24253,25005..25436))	Correct gene location
	rpoC2	complement(18299..22453)	Correct gene location
	rps19	join(162917..162962,1..3)	Re-annotate a missing gene in IRa
	ycf1	116235..117482	Correct gene location
	ycf15	join(100165..100318,100611..100715)	Re-annotate a missing gene in IRb
	ycf15	complement(join(153148..153252,153545..153698))	Re-annotate a missing gene in IRa
Hovenia trichocarpa MT225404	*atpF*	complement(14382..14527),complement(13647..13237)	Re-annotate a missing gene in LSC
	ndhD	complement(123528..122017)	Re-annotate a missing gene in SSC
	petD	join(81631..81637,82375..82850)	Re-annotate a missing gene in LSC
	psbC	36669..38090	Re-annotate a missing gene in LSC
	psbL	complement(68668..68552)	Re-annotate a missing gene in LSC
	rpoC2	complement(18201..22364)	Correct gene location
	rps19	complement(89587..89309)	Re-annotate a missing gene in IRb
	rps19	join(161537..161668,1..12)	Re-annotate a missing gene in IRa
	trnfM-CAU	39783..39856	Re-annotate a missing gene in LSC

Species	Gene	Updated position	Detail
	trnG-UCC	join(9937..9959,10664..10712)	Re-annotate a missing gene in LSC
	ycf1	complement(130511..136267)	Correct gene location
	ycf1	114857..116092	Re-annotate a missing gene in IRb
	ycf15	join(98789..98942,99235..99339)	Re-annotate a missing gene in IRb
	ycf15	complement(join(151785..151889,152182..152335))	Re-annotate a missing gene in IRa
Rhamnus crenata LC635131	*psbC*	35926..37347	Correct gene location
	rpoC1	complement(join(21728..23338,24122..24553))	Correct gene location
	trnL-UAG	129617..129696	Re-annotate a missing gene in SSC
	trnN-GUU	113774..113844	Re-annotate a missing gene in IRb
	trnN-GUU	135495..135565	Re-annotate a missing gene in IRa
	ycf1	complement(134037..135165)	Re-annotate a missing gene in IRa
	ycf15	join(98082..98235,98512..98616)	Re-annotate a missing gene in IRb
	ycf15	complement(join(150723..150827,151104..151257))	Re-annotate a missing gene in IRa
Rhamnus globosa MT360052	*ndhD*	complement(121205..122716)	Correct gene location
	ndhF	complement(115291..117543)	Correct gene location
	psbL	complement(68312..68196)	Re-annotate a missing gene in LSC
	rps19	join(160543..160642,1..2)	Re-annotate a missing gene in IRa

Species	Gene	Updated position	Detail
	ycf15	join(98175..98328,98605..98709)	Re-annotate a missing gene in IRb
	ycf15	complement(join(150823..150927,151204..151357))	Re-annotate a missing gene in IRa
Rhamnus heterophylla MT211599	*ndhD*	complement(117369..118880)	Correct gene location
	psbL	complement(64386..64502)	Correct gene location
	rps19	join(156415..156514,1..2)	Re-annotate a missing gene in IRa
	ycf1	111037..111336	Re-annotate a missing gene in IRb
	ycf15	join(95304..95457,95886..95990)	Re-annotate a missing gene in IRb
	ycf15	complement(join(146548..146652,147081..147234))	Re-annotate a missing gene in IRa
Rhamnus taquetii NC_045855	*ndhD*	complement(121710..123221)	Correct gene location
	psbL	complement(68653..68769)	Correct gene location
	rps19	join(161106..161205,1..2)	Re-annotate a missing gene in IRa
	ycf1	114737..115825	Re-annotate a missing gene in IRb
	ycf15	join(98654..98807,99084..99188)	Re-annotate a missing gene in IRb
	ycf15	complement(join(151391..151495,151772..151925))	Re-annotate a missing gene in IRa
Spyridium parvifolium MH234313	*ndhD*	complement(121535..123046)	Correct gene location
	psbL	complement(67924..68090)	Correct gene location
	rpoC2	complement(17998..22161)	Correct gene location
	rps19	160786..160911	Re-annotate a missing gene in IRa

Species	Gene	Updated position	Detail
	trnV-UAC	join(54583..54619,55216..55254)	Re-annotate a missing gene in LSC
	ycf1	113993..115210	Re-annotate a missing gene in IRb
	ycf15	join(97936..98089,98365..98469)	Correct gene location
	ycf15	complement(join(151116..151220,151496..151649))	Correct gene location
Ventilago leiocarpa MT974496	*infA*	complement(85540..85726)	Re-annotate a missing gene in LSC
	rps19	complement(90251..89973)	Re-annotate a missing gene in IRb
	rps19	161693..161809	Re-annotate a missing gene in IRa
	ycf1	complement(136527..130763)	Re-annotate a missing gene in IRa
	ycf1	115417..116427	Re-annotate a missing gene in IRb
	ycf15	join(99399..99552,99829..99933)	Re-annotate a missing gene in IRb
	ycf15	complement(join(152011..152115,152392..152545))	Re-annotate a missing gene in IRa
Ziziphus incurva NC_050251	*ndhD*	complement(121369..122880)	Correct gene location
	ndhE	complement(123503..123808)	Correct gene location
	psbJ	complement(67962..68084)	Correct gene location
	psbL	complement(68327..68267)	Correct sequence
	rpoC1	complement(join(22273..23907,24681..25112))	Correct gene location
	rpoC2	complement(17962..22119)	Correct gene location
	rps19	join(160814..160920,1..22)	Re-annotate a missing gene in IRa

Species	Gene	Updated position	Detail
	ycf1	114129..115274	Re-annotate a missing gene in IRb
	ycf15	join(98073..98226,98519..98623)	Re-annotate a missing gene in IRb
	ycf15	complement(join(151076..151180,151473..151626))	Re-annotate a missing gene in IRa
Ziziphus jujuba KX266829	*accD*	61291..63024	Correct gene location
	ndhD	complement(121720..123231)	Correct gene location
	ndhE	complement(124159..123854)	Re-annotate a missing gene in SSC
	psbL	complement(68044..68160)	Correct gene location
	rpoC2	complement(18047..22204)	Correct gene location
	rps19	join(161109..161215,1..28)	Re-annotate a missing gene in IRa
	ycf15	join(98227..98380,98673..98777)	Re-annotate a missing gene in IRb
	ycf15	complement(join(151413..151517,151810..151963))	Re-annotate a missing gene in IRa
Ziziphus jujuba KX266830	*accD*	61290..63023	Correct gene location
	ndhD	complement(121677..123188)	Correct gene location
	ndhE	complement(124116..123811)	Re-annotate a missing gene in SSC
	psbL	complement(68018..68134)	Correct gene location
	rpoC2	complement(18018..22175)	Correct gene location
	rps19	join(161105..161211,1..28)	Re-annotate a missing gene in IRa
	ycf15	join(98192..98345,98638..98742)	Re-annotate a missing gene in IRb
	ycf15	complement(join(151367..151471,151764..151917))	Re-annotate a missing gene in IRa

Species	Gene	Updated position	Detail
Ziziphus jujuba MF781071	*infA*	complement(84710..84769)	Re-annotate a missing gene in LSC
	psbN	complement(78973..79104)	Correct gene location
	rps12	complement(join(74178..74291,103076..103051,103845..103614))	Re-annotate a missing gene in IRb
	rps12	join(complement(74178..74291),146827..147058,147596..147621)	Correct gene location
	rps19	join(161387..161493,1..88)	Re-annotate a missing gene in IRa
	ycf1	114527..115666	Re-annotate a missing gene in IRb
	ycf15	join(98474..98627,98920..99024)	Re-annotate a missing gene in IRb
	ycf15	complement(join(151649..151753,152046..152199))	Re-annotate a missing gene in IRa
Ziziphus jujuba MW160433	*accD*	61314..63047	Correct gene location
	ndhD	complement(122071..123582)	Correct gene location
	ndhE	complement(124510..124205)	Re-annotate a missing gene in SSC
	psbL	complement(68413..68529)	Correct gene location
	rpoC2	complement(18032..22189)	Correct gene location
	rps19	join(161500..161606,1..28)	Re-annotate a missing gene in IRa
	ycf15	join(98587..98740,99033..99137)	Correct gene location
	ycf15	complement(join(151762..151866,152159..152312))	Correct gene location
Ziziphus jujuba NC_030299	*infA*	complement(84650..84709)	Re-annotate a missing gene in LSC
	rpoC2	complement(17849..22006)	Correct gene location

Species	Gene	Updated position	Detail
	rps19	join(161360..161466,1..22)	Re-annotate a missing gene in IRa
	trnV-UAC	join(54936..54972,55569..55607)	Re-annotate a missing gene in LSC
	ycf1	complement(130377..136101)	Re-annotate a missing gene in IRa
	ycf1	114486..115640	Re-annotate a missing gene in IRb
	ycf15	join(98433..98586,98879..98983)	Correct gene location
	ycf15	complement(join(151604..151708,152001..152154))	Correct gene location
Ziziphus mauritiana NC_037151	*accD*	61232..62959	Correct gene location
	ndhD	complement(121938..123449)	Correct gene location
	ndhE	complement(124378..124073)	Re-annotate a missing gene in SSC
	psbL	complement(68303..68419)	Correct gene location
	rpoC2	complement(17892..22049)	Correct gene location
	rps19	join(161327..161543,1..23)	Re-annotate a missing gene in IRa
	ycf1	114513..115658	Re-annotate a missing gene in IRb
	ycf15	join(98468..98621,98914..99018)	Re-annotate a missing gene in IRb
	ycf15	complement(join(151607..151711,152004..152157))	Re-annotate a missing gene in IRa
Ziziphus spina-christi NC_037152	*accD*	61286..63031	Correct gene location
	ndhD	complement(122011..123522)	Correct gene location
	ndhE	complement(124451..124146)	Re-annotate a missing gene in SSC

Species	Gene	Updated position	Detail
	psbL	complement(68359..68475)	Correct gene location
	rpoC2	complement(17923..22080)	Correct gene location
	rps19	join(161399..161615,1..23)	Re-annotate a missing gene in IRa
	ycf1	114593..115738	Re-annotate a missing gene in IRb
	ycf15	join(98548..98701,98994..99098)	Re-annotate a missing gene in IRb
	ycf15	complement(join(151679..151783,152076..152229))	Re-annotate a missing gene in IRa
Cannabis sativa NC_027223*	*ycf15*	93652..93728	Re-annotate a missing gene in IRb
	ycf15	complement(144154..144227)	Re-annotate a missing gene in IRa
Ficus religiosa NC_033979*	*ndhD*	complement(121639..123150)	Correct gene location
	ndhK	complement(53441..54121)	Correct gene location
	psbL	complement(68539..68423)	Re-annotate a missing gene in LSC
	rbcL	58910..60337	Correct gene location
	rpoC2	complement(17877..22040)	Correct gene location
	rps12	join(146365..146596,147133..147158,complement(74189..74302))	Re-annotate a missing gene in IRa
	rps19	join(160535..160627,1..6)	Re-annotate a missing gene in IRa
	ycf15	98148..98252	Correct gene location
	ycf15	complement(151191..151295)	Correct gene location

* Outgroups in the phylogeny

Table S4. List of orthologous genes for phylogenetic reconstruction.

**Function**	**Gene list**
ATP-dependent protease proteolytic subunit	*clpP1*
ATP synthase	*atpA, atpB, atpE, atpF, atpH, atpI*
Cytochrome b/f complex	*petA, petB, petD, petG, petL, petN*
Cytochrome complex assembly	*ccsA*
Envelope membrane protein	*cemA*
Hypothetical chloroplast reading frames	*ycf1(IRa), ycf2*
Maturase	*matK*
NADH dehydrogenase	*ndhA, ndhB, ndhC, ndhD, ndhE, ndhF, ndhG, ndhH, ndhI, ndhJ, ndhK*
Photosystem I	*psaA, psaB, psaC, psaI, psaJ, ycf3, ycf4*
Photosystem II	*psbA, psbB, psbC, psbD, psbE, psbF, psbH, psbI, psbJ, psbK, psbL, psbM, psbN, psbT, psbZ*
RubisCO large subunit	*rbcL*
Subunit of acetyl-CoA-carboxylase	*accD*
Large subunit of ribosomal proteins (LSU)	*rpl14, rpl16, rpl2, rpl20, rpl22, rpl23, rpl32, rpl33, rpl36*
Small subunit of ribosomal proteins (SSU)	*rps2, rps3, rps4, rps7, rps8, rps11, rps12, rps14, rps15, rps16, rps18, rps19 (IRb)*
RNA polymerase	*rpoA, rpoB, rpoC1, rpoC2*
